# Seed Moisture Isotherms, Sorption Models, and Longevity

**DOI:** 10.3389/fpls.2022.891913

**Published:** 2022-06-02

**Authors:** Fiona R. Hay, Shabnam Rezaei, Julia Buitink

**Affiliations:** ^1^Department of Agroecology, University of Aarhus, Slagelse, Denmark; ^2^Université d'Angers, Institut Agro, INRAE, IRHS, SFR QUASAV, Angers, France

**Keywords:** equilibrium relative humidity (eRH), isotherm, moisture content, storage stability, seed longevity

## Abstract

Seed moisture sorption isotherms show the equilibrium relationship between water content and equilibrium relative humidity (eRH) when seeds are either losing water from a hydrated state (desorption isotherm) or gaining water from a dry state (adsorption isotherm). They have been used in food science to predict the stability of different products and to optimize drying and/or processing. Isotherms have also been applied to understand the physiological processes occurring in viable seeds and how sorption properties differ in relation to, for example, developmental maturity, degree of desiccation tolerance, or dormancy status. In this review, we describe how sorption isotherms can help us understand how the longevity of viable seeds depends upon how they are dried and the conditions under which they are stored. We describe different ways in which isotherms can be determined, how the data are modeled using various theoretical and non-theoretical equations, and how they can be interpreted in relation to storage stability.

## Introduction

Seeds are both an agricultural input and an agricultural output. As an input, they are produced in one season and stored for planting in a subsequent season. Storage may be in on-farm stores or, in the shorter term, in a company warehouse, before they are distributed and sold for sowing. As a means of conserving and sharing different varieties, seeds are also stored in community seed banks or in genebanks (Westengen et al., 2018). As an output, seeds are stored as a product for both human and animal consumption, as grain in the case of cereals, but also prior to extraction of constituents such as oils. How the seeds are stored will depend on their intended use and the length of time they need to remain in a usable state. For use in planting, seeds will be stored such that an acceptable level of physiological quality (viability, vigor) will be maintained over the expected storage period. As a commodity, seeds (“grain” in the case of cereal seeds) will be stored to avoid losses due to, for example, insects, fungi and biochemical reactions (Mahapatra and Lan, 2007; Sultana et al., 2021; Ziegler et al., 2021). The actual storage conditions will depend on the expected storage period, i.e., how long the seeds need to be stored to meet demand before new seeds are produced.

Regardless of the intended use of the seeds, the three most important external factors that determine how long they can be stored before the quality declines to unacceptable levels are the moisture content, the temperature and the gaseous environment, and scientists from different fields have attempted to describe how these factors influence storability (e.g., Roberts and Ellis, 1989; Walters et al., 2005; Rahman and Labuza, 2007; Buitink and Leprince, 2008; Ziegler et al., 2021). In the food science literature, moisture sorption isotherms are often used to understand the stability of foods in different environments (e.g., Staniszewska et al., 2020; Rosa et al., 2021); they have also been used to understand seed physiology, particularly in relation to seed storage (e.g., Sun et al., 1997; Hay and Timple, 2016). However, the approaches to construct and interpret seed isotherms differ, as seed scientists are focused on the viability of the seeds, which is generally of little or no interest to food scientists. In this review, we explore how seed isotherms have been measured, interpreted and used in the literature, particularly in relation to understanding the role of moisture, and its dependence on temperature, in the storability of seeds as both an agricultural input and an agricultural output. We explain the different experimental methodologies used to measure those isotherms, the various models that have been used to fit moisture sorption data, and discuss how seed moisture sorption isotherms can be used as a tool to predict the types and rates of biochemical reactions that can occur in seeds.

## What Are Moisture Sorption Isotherms and Why Are They Informative?

A moisture sorption isotherm is the relationship, when the system is in equilibrium at a specific temperature, between moisture content (usually expressed as a proportion or percentage of the dry weight, though percentage fresh weight is common in the seed science literature) and relative humidity (RH) or water activity (*a*_w_, where *a*_w_ ≅ RH/100) (Bell and Labuza, 2000; Basu et al., 2006). Water activity is defined as the (water) vapor pressure of a sample relative to the vapor pressure of pure water and indicates the availability of water to promote aqueous reactions (Vertucci and Roos, 1993; Chirife and Fontana, 2020). In the food literature, isotherms became of interest with the realization that, in relation to storage stability, the amount of water in a food is less important than the *a*_w_ of the water (Rahman and Labuza, 2007). That is, the type of reactions and relative rate of reactions for different types of foods were better related with *a*_w_ than with moisture content. Thus, in the food literature, storage experiments often refer to *a*_w_ or storage RH (e.g., Fu et al., 2020; Chen et al., 2021; Zhao et al., 2021), and/or involve the determination of isotherms (e.g., Staniszewska et al., 2020; Rosa et al., 2021). When the temperature of the sample is below the relevant glass transition threshold (see below), reliable detection of *a*_w_ is not possible, while an apparent RH may still be detected (Schiraldi et al., 2012). Thus, hereafter in discussing seed isotherms, we will use the term equilibrium relative humidity (eRH) meaning the RH of the air around the seeds, either when measured (i.e., of the air within the sample chamber at equilibrium) or being the RH of the equilibration environment, unless discussing the food literature where *a*_w_ is the parameter used.

**Figure 1:**
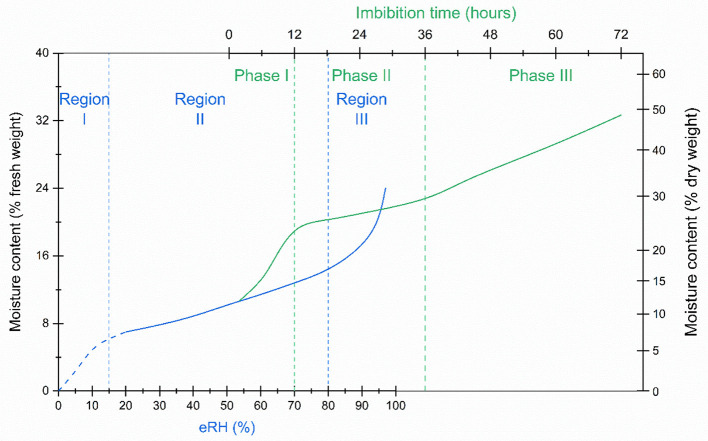
A seed isotherm (blue line), the relationship between moisture content and water activity, is typically sigmoidal, with three regions (region I, II and III in blue text) indicative of the how the water is bound within the seed tissues. The imbibition curve (green line), which follows the change in seed moisture content over time, is also tri-phasic (phase I, II and III in green text). Seeds are usually “air-dry”, typically within region II of the seed isotherm, when they are sown. Water uptake which takes the seeds into region III of the isotherm, is initially fast, but is then more or less constant until radicle emergence at the end of phase II of the imbibition curve. Water content increases in phase III as the seedling develops. If the seeds have dried to a lower moisture level, approaching or within region I of the isotherm, rapid influx of liquid water may result in irreparable damage to the cell membranes. To avoid such imbibition injury, rehydration in a humid atmosphere, for example, over water, is recommended (Bewley et al., 2013). This schematic is broadly based on data for rice seeds from Hay and Timple (2016) and Zhao et al. (2020).

Moisture sorption isotherms are categorized according to their specific shape, reflecting the kinetic and thermodynamic properties of the water molecules (Brunauer et al., 1938; Al-Muhtaseb et al., 2002; Caballero-Cerón et al., 2015; Okos et al., 2019). Many orthodox seeds and food products, show a type II isotherm which is sigmoidal (Al-Muhtaseb et al., 2002) (Figure 1). In the literature, type II isotherms have been divided into regions: in region I, most of the water is strongly bound at hydrophilic, charged and polar groups; in region II, water molecules become predominantly bound to hydrophilic sites and act as a solvent for some reactions; and in region III, in addition to the tight- and weakly-bound water, more and more water is multi-molecular or free water associated with other water molecules (Walters et al., 2002, 2005; Okos et al., 2019). Region III may be further subdivided into regions III, IV, and V (Walters et al., 2002, 2005). The isotherm should not be confused with the tri-phasic pattern of water imbibition by seeds, which has time on the *x*-axis and typically starts somewhere in region II of the isotherm, depending on the initial moisture content prior to imbibition (Figure 1). In seeds without physical dormancy (i.e., that do not have a water impermeable seed coat), there is relatively rapid uptake of water during Phase I of the imbibition curve, whereas during phase II, seed moisture content is relatively stable. The boundary between phases II and III is when the radicle emerges from the seed (germination *sensu stricto*), after which moisture content increases with seedling growth (Bewley et al., 2013).

**Figure 2:**
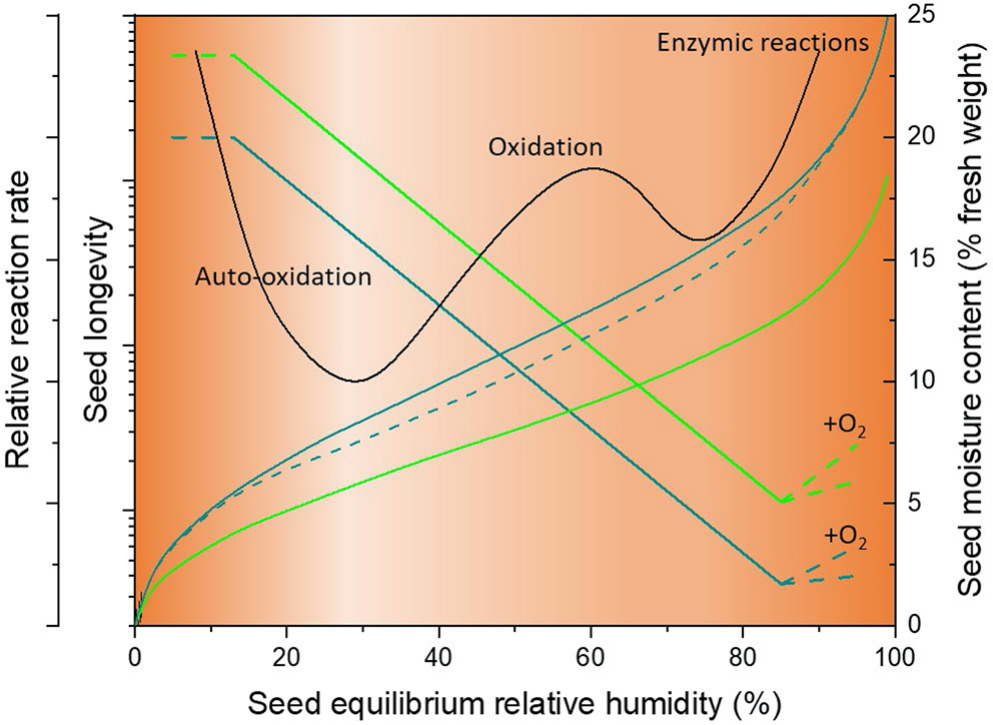
Schematic showing relative reaction rate (food stability diagram), seed longevity and moisture content plotted against equilibrium relative humidity. The sigmoidal teal and green solid sigmoidal lines illustrate desorption isotherms for barley and lettuce seeds, respectively (with moisture content as right *y*-axis). The dashed teal curve illustrates the adsorption isotherm (for barley seeds only), showing the effect of hysteresis. The solid straight lines indicate the relative longevity (left *y*-axis) of barley (teal) and lettuce (green) seeds in hermetic storage based on Roberts and Ellis (1989). At very high eRH, when oxygen is freely available (+O_2_) longevity increases due to macromolecular repair (Figure 1). The black zig-zag curve shows the food stability isotherm, with relative reaction rate for aging reactions on the leftmost *y*-axis and an indication of the types of reactions occuring in the presence of oxygen (Rahman and Labuza, 2007). The orange shading also reflects this stabilty diagram, with lighter shading indicating greater stability.

In food science, isotherms are primarily used to understand the effects of drying, processing and storage on the quality of food products (Supplementary Table 1). In particular, by fitting a theoretical model such as the Brunauer-Emmett-Teller (BET) or Gugenheim-Anderson-de Boer (GAB) (see below), they are often used to determine what is known as the “monolayer moisture content” (*M*_m_). In theory, as moisture content increases above *M*_m_ at higher eRH, more and more water becomes available to act as a solvent and thereby facilitate reactions (Bell and Labuza, 2000). Since ionic-bound water cannot participate in chemical reactions, *M*_m_ is considered the point at which product stability is maximized (Caballero-Cerón et al., 2015). The *M*_m_ occurs over a broad range of moisture contents depending on the composition of the food being considered (1–14% dry weight basis), but typically lies between 0.2 and 0.4 on the *a*_w_ scale (Rahman and Labuza, 2007). Since the isotherms shift to lower moisture contents at the same *a*_w_ as temperature is increased, the *M*_m_ value decreases as temperature increases (e.g., Ayranci and Duman, 2005; Talla, 2014). In the food science literature, the isotherm is often overlaid by a stability diagram, illustrating the type and relative rate of spoilage that occurs in foods at different *a*_w_, including the growth of yeast and bacteria at high *a*_w_ (>0.75), oxidative reactions such as Maillard reactions which peak at 0.6–0.7 *a*_w_, and auto-oxidative reactions at *a*_w_ below *M*_m_ (Rahman and Labuza, 2007; Okos et al., 2019) (Figure 2).

**Figure 3:**
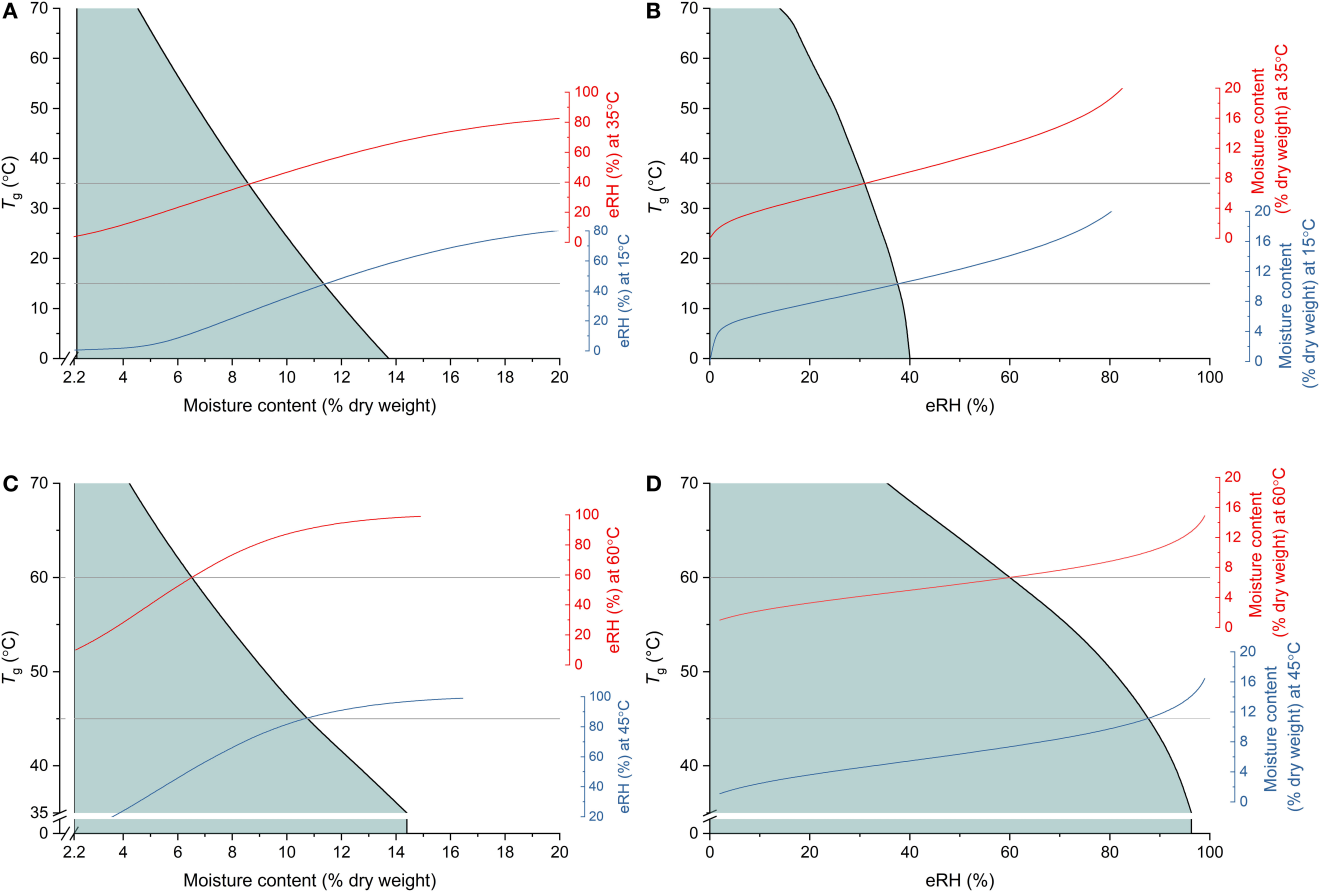
Combining the concepts of glass transition theory and isotherm modeling to understand storage stability. **(A,C)** Show the state diagrams (glass transition temperature [*T*_g_ ] vs. moisture content) for cotyledon slices of pea cotyledons (from Buitink et al., 1999) and sunflower seeds (from Lehner et al., 2006), respectively, with the shaded area indicating the temperature-moisture content region where the tissue would be in a glassy state. Also shown are isotherms based on **(A)** fitted equations from Vertucci and Leopold (1987) at 15 and 35°C or **(C)** Cromarty's equation (Cromarty et al., 1982) at 45 and 60°C. The horizontal gray lines indicate these respective temperatures and the isotherms are positioned such that they meet *T*_g_ at the corresponding moisture content. The same relations are shown in **(B,D)**, respectively, plotted against eRH rather than moisture content. It should be noted, however, that these glass transition temperatures were determined using differential scanning calorimetry in which samples are not in equilibrium and hence actual relationships between *T*_g_ and eRH (and be extension, moisture content) are not known.

Understanding the shelf-life of foods is not limited to the framework of moisture sorption isotherms; state diagrams are also used to map the physical state of foods according to their moisture content (usually plotted as the *x*-axis) and temperature (*y*-axis) (see Rahman, 2007 and references therein, e.g., Staniszewska et al., 2020; Kyomugasho et al., 2021). Similarly, state diagrams for seeds and/or different seed tissues have been published in the plant/seed science literature (e.g., Leopold et al., 1994; Sacande et al., 2000; Pukacka et al., 2003; Matiacevich et al., 2006). The primary transition plotted in a state diagram is the glass transition temperature, *T*_g_. At temperatures and/or moisture contents below the *T*_g_ curve, materials would be in a glassy state where molecular mobility is restricted and hence, aging reactions slowed (Leopold et al., 1994; Buitink and Leprince, 2008; Ballesteros and Walters, 2019) (Figure 3). Thus, while an isotherm can indicate the type of reactions that might be occurring, and to some extent the rate of those reactions, the rate can be further explained by whether or not the material is in a glassy state. Nonetheless, it is recognized that the connection between these two stability concepts has not often been made (Rahman, 2007; Chigwedere et al., 2019) and there are surprisingly few papers reporting both aspects of food stability. This may be because of the different temperature ranges over which they are determined; isotherms will typically be determined over more ambient temperatures (e.g., 5–50°C), while the calorimetric method used to determine transitions, lends itself to extending to much lower, sub-zero temperatures. Further, the *T*_g_ of seeds has most often been determined using differential scanning calorimetry (DSC) in which the scan rate may be, e.g., 10–20°C min^−1^, i.e., when the seed tissue being tested, albeit a small amount (e.g., “*a few to tens of milligrams*”; Williams, 1994), is unlikely to be in equilibrium. Consequently, it is difficult to directly relate the *T*_g_ to a particular eRH and, moreover, it suggests that the *T*_g_-moisture content relationship should be interpreted with some caution (Figure 3). Nonetheless, it has been reported that the moisture content at which the glass transition occurs decreases as temperature increases (Figure 3), as does *M*_m_. But, it is hard to directly relate *M*_m_ with *T*_g_. Some examples where both isotherms and transitions diagrams were determined are found in Ballesteros and Walters (2011) and in Buitink et al. (1998). Buitink et al. (1998) showed that the *M*_m_ of pea axes or *Typha latifolia* pollen was at lower moisture contents/temperatures than the glass transition temperature, i.e., *M*_m_ occurred when the cytoplasm was, according to DSC, in a glassy state.

Nonetheless, understanding of both the moisture content-*a*_w_ relationship and *T*_g_ are considered important in relation to drying foods. Specifically, it is recommended that food products are stored at *M*_m_ for maximum stability, but kept above *T*_g_ during the drying process to ensure efficient movement of water out of the food. This is because once the material enters a glassy state, mobility is reduced and hence drying will be slowed (Okos et al., 2019). This has implications in relation to the genebank standards for drying, where a low temperature is recommended for drying (5–20°C and 10–25% RH; FAO, 2014). Drying at low temperatures means that the cytoplasm of seeds will go in to a glassy state at a higher moisture content (and eRH), slowing the drying rate further and prolonging the time when seeds are at a high moisture content and therefore at risk of aging. It can be surmised that a two-stage drying process, separating the “active drying” from the “final equilibrium drying” has the potential to be far more efficient. Initial drying at a higher temperature will mean the seeds will dry faster and reach *T*_g_ both more quickly and at a lower moisture content. The final, slower equilibrium drying to the target moisture content for long-term seed storage, which is typically lower than that the *M*_m_ used by food scientists, can then be done at a lower temperature. Such a protocol has been shown to be beneficial to the subsequent longevity of rice seeds harvested at high moisture content (Whitehouse et al., 2015, 2018).

## Influence of Composition, Temperature and Moisture History on the Shape of the Isotherm

**Figure 4:**
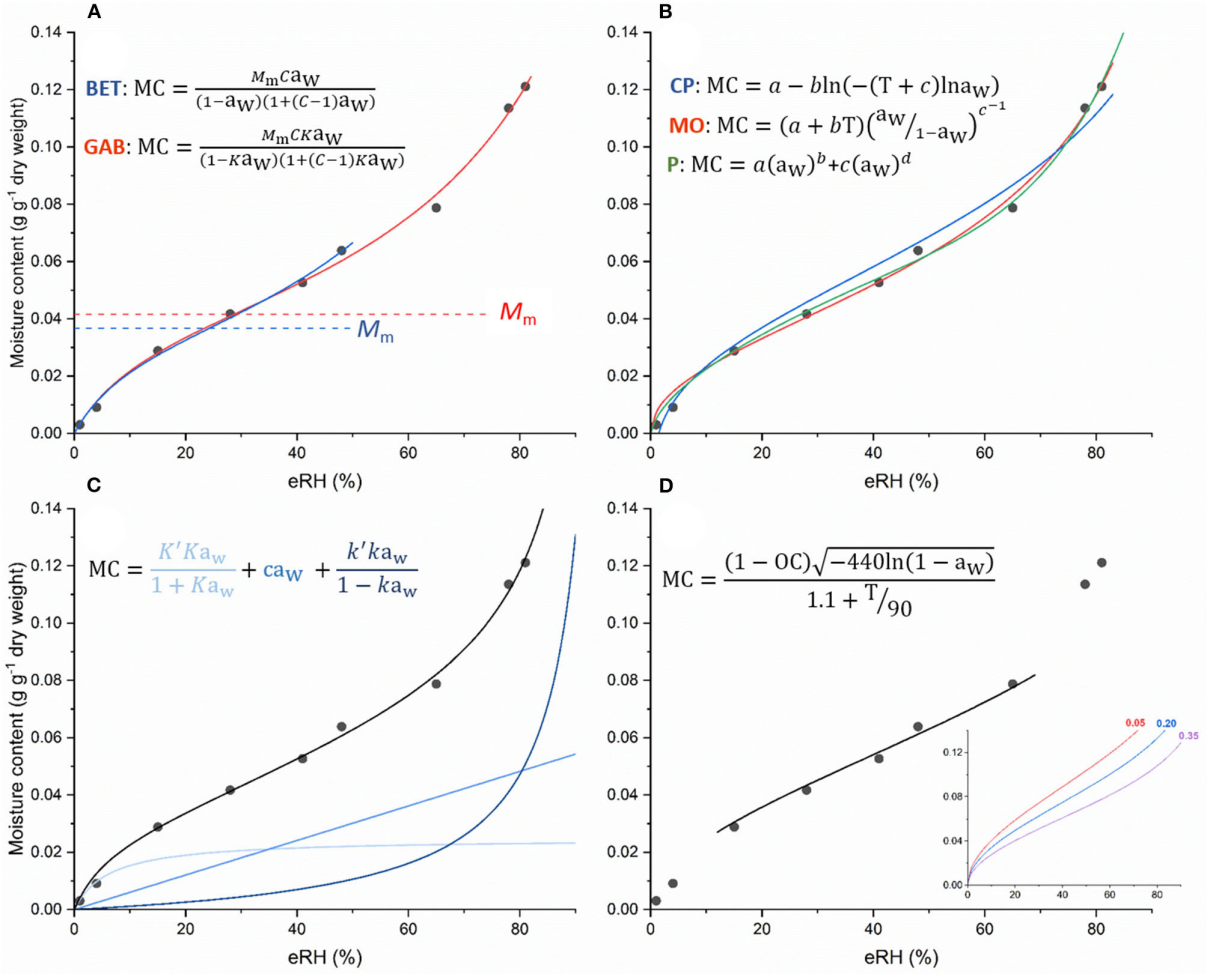
Examples of different equations fitted to isotherm data [moisture content vs. equilibrium relative humidity (eRH)] from Hay et al. (2003) for seeds of *Arabidopsis thaliana* at 45°C. In the various equations MC is the moisture content and in isotherm equations, is usually expressed as g g^−1^ dry weight (see Figure 1 for conversions between fresh and dry weight basis); *a*_w_ is the water activity (where *a*_w_ ≅ eRH/100, but see text); *T* is temperature (°C); OC is oil content; and letters in italics are parameters for estimation (variously, *M*_m_, *C, a, b, c, K*′, *K*, *k*′ and *k*, according to convention in the literature). Equations shown in **(A,C)** represent theoretical models, in **(A)**, the Brunauer-Emmett-Teller (BET) and Gugenheim-Anderson-de Boer (GAB) equations and in **(C)**, the D'Arcy-Watt equation. The D'Arcy-Watt equation has three terms with parameters (*K*, *K*′, *c, k*, *k*′) that can be interpreted as representing the number and strength of strong (light blue), weak (mid-blue) and multi-molecular (dark blue) water binding sites, respectively. One of the parameters estimated when fitting the BET and GAB equations, is the monolayer value, *M*_m_, and differences in the estimates from the two models are apparent in **(A)**. This monolayer value is also different than the amount of water that is strongly bound as determined by fitting the D'Arcy-Watt equation **(C)**. **(B)** shows the results of fitting various (semi-) empirical equations: Chung-Pfost (CP), modified-Oswin (MO) and Peleg (P). As well as taking different forms, it can be seen how the fit is improved when there are more parameters in the equation (the Peleg equation has four, whereas the other two equations have two). In **(D)**, the results of fitting Cromarty's equation to the same data (limited *a*_w_ range) is shown, with oil content (OC) as a parameter that is estimated. This is not how Cromarty's equation is normally used. Rather, it is used to predict the moisture content after drying at a particular relative humidity and temperature, depending on seed oil content. This equation illustrates how the isotherm shifts to lower moisture contents as oil content increases [inset graph, for seeds with oil contents of 0.05, 0.20, and 0.35 (dry weight basis); axes as for main graph].

While seeds and foods often have what is described as a type II isotherm that is sigmoidal in shape (Figure 2), the actual shape and “height” (i.e., range covered on the moisture content scale) depends on composition and temperature. In terms of composition, for heterogeneous materials such as seeds, the amount of oil they contain largely determines how much water will be adsorbed, as reflected in Cromarty's equation which includes oil content as a variable (Cromarty et al., 1982; see below and Figure 4D). The lower the oil content, the greater the amount of water that will be adsorbed, as seen for example for seeds of *Camellia oleifera* with differing oil content (Zhu et al., 2021). This is a key reason why equilibrating seeds so they have the same eRH can be considered preferable to equilibrating seeds to the same moisture content when comparing physiological responses such as seed longevity across diverse species (Hay et al., 2022). It also explains why different tissues within a seed can have different moisture contents. For example, the storage reserve tissues (cotyledons) of seeds of neem (*Azadirachta indica*; Sacande et al., 2000) have a higher oil content than the embryonic axis, and therefore, at equilibrium (i.e., when eRH is uniform throughout the seed), the moisture content of the embryonic axis is higher. Although oil content largely determines the amount of water a seed might adsorb at a given RH, the kinetics and thermodynamics of the water within the seed tissues will nonetheless be affected by other macromolecules. For instance, starch has a higher hydration capacity than protein, and even the amino acid compositions of proteins affect their hydration properties (Kuntz and Kauzmann, 1974); thus both the amount and type of proteins present in seeds will influence the moisture content-eRH relationship, as will sugars (Wang et al., 2009). As such, it can be expected that there will be subtle differences in seed isotherms among different crop varieties, especially if varieties are selected based on seed composition.

Temperature also modifies the shape of the isotherm. As temperature is reduced, for seeds with a given eRH, the moisture content is higher; or conversely, as temperature is reduced, at a given moisture content, the eRH is lower (Vertucci and Roos, 1993; Fang et al., 1998; Kartika et al., 2012; Zeymer et al., 2017; Arslan-Tontul, 2020; Zhu et al., 2021) (Supplementary Figure 1). Again, this effect tends to be greater over the mid-eRH-range, as seen for example, for seeds of grape (Maleki Majd et al., 2013), rice (Mousa et al., 2014) and maize (Talla, 2014), though this pattern was less apparent in the isotherms determined for seeds of pea, soya bean and peanut by Vertucci and Roos (1993). Isotherms measured at different temperatures allow calculation of the isosteric heat of sorption using the Clausius-Clapeyron equation (Quirijns et al., 2005; Chen, 2006). This heat of sorption is indicative of the strength of bonding forces of the water molecules and helps to explain drying kinetics: at high eRH, a lot of the water molecules in the tissues are “multimolecular” or “free” water (Figure 1) and therefore are removed from the seeds more easily compared with water molecules that are ionically bound at low eRH. To remove these ionically bound water molecules, more energy needs to be put into the system. This explains why heat is used to dry seeds for moisture content determination (ISTA, 2022).

The shape of the isotherm is also highly dependent on whether the seeds are taking up moisture or losing moisture, a phenomenon known as “hysteresis”, and thus desorption and adsorption isotherms can be defined (Bell and Labuza, 2000; Schiraldi et al., 2012) (Figure 2; Supplementary Figure 1). The desorption isotherm gives higher moisture contents at a particular eRH than the adsorption isotherm, particularly over the middle region of the eRH scale (20–80%) (Yang et al., 1997; Raji and Ojediran, 2011; Mousa et al., 2014; Mallek-Ayadi et al., 2020). As such, the isosteric heat is greater for seeds that are losing moisture, than for seeds that are gaining moisture, though this effect is reduced at higher moisture contents (Li et al., 2011; Mousa et al., 2014). If the material has already been dried to an intermediate eRH, then equilibrating seeds at a wide range of relative humidities means that the “working isotherm” (Bell and Labuza, 2000) may be a combination of both the ad- and desorption isotherms.

In the case of seeds, the first desorption isotherm, determined for seeds directly from the field (see e.g., Whitehouse et al., 2015) is probably most interesting in terms of relating the results to seed drying prior to storage. However, it is perhaps more common for pre-dried seeds to be used. Seed storage experiments, for example, tend to be conducted for seeds that are adsorbing, since the moisture content is normally increased so that loss of viability can be followed over a reasonable length of time (Ellis and Roberts, 1980a,b; Newton et al., 2014; De Vitis et al., 2020; Hay et al., 2022). According to Rahman and Labuza (2007), “*the desorption hysteresis loop usually ends at the monolayer*” or, in other words, that it is necessary to dry below *M*_m_ for the material to cross over to the adsorption isotherm when water is added. However, Hay and Timple (2016) found that rice seeds switched to the adsorption isotherm upon rehydration, even after drying to just 10% moisture content (fresh weight basis; approximately 45% eRH). Furthermore, Hay and Timple (2016) found, in contrast to what the food literature might suggest, that the moisture content was more predictive of longevity than seed eRH. Bello et al. (2011) similarly reported that viability loss was similar for desorping and adsorping seeds at the same moisture content rather than relative humidity. That is, that adsorping seeds stored at a particular eRH had a lower moisture content and therefore, rate of viability loss, than desorping seeds at the same eRH. This is strikingly inconsistent with the food literature in which *a*_w_ is considered more relevant than moisture content, but could perhaps be related to the fact that seed scientists are focused on the biological impact of seed aging rather than the aging of inert material.

Repeated cycles of de- and adsorption may shift the isotherms away from the first original de- and adsorption isotherms, depending on the temperature (Okos et al., 2019). Li et al. (2011) for example, reported differences in the desorption isotherms of freshly-harvested wheat seeds and seeds that had been previously dried (to less than 5%, i.e., to below the monolayer water content value determined from fitting the BET equation) and then rewetted.

There are various hypotheses as to why hysteresis occurs; one suggestion is that when seeds have been dried, the capillaries do not fill up completely when they subsequently take up moisture, hence the isotherm is shifted to lower moisture contents (Wolf et al., 1972; Rahman and Al-Belushi, 2006). In terms of seed science, it perhaps suffices to say that knowing and reporting information about the source of seeds used in experiments, including moisture history, is important when comparing results across experiments to improve our understanding of seed physiological responses. However, there is an implication for when we use environmental parameters to predict moisture content using Cromarty's equation (Cromarty et al., 1982; see below) and then use the predicted moisture content in the viability equations to predict longevity (Box 1). Indeed, as well as moisture history, temperature history may also influence moisture sorption behavior. Hay et al. (2013) reported differences in the moisture content-eRH relationship at 22–22.5° of rice seeds that had been previously stored at −10 then −20°C vs. 2–4°C for 22–32 years. Although seeds had been dried and vacuum-packed at the same time, the data suggested that the isotherm for seeds stored at 2–4°C was lower (i.e., lower moisture content at the same eRH = higher eRH at the same moisture content) for seeds that had been stored at 2–4°C.

Box 1 **The seed viability equations describe the decline in viability during storage of seeds under controlled, hermetic conditions (constant moisture content and temperature).**

For each individual seed lot in a particular storage environment, a survival curve can be described according to the equation:

$$\begin{array}{l} v=K_{i}-\frac{p}{\sigma } \end{array}$$

where *v* is the viability in units of normal equivalent deviates (NED) (=probits, which can be transformed to a percentage value), *K*_i_ is the initial viability (NED), *p* is the storage period and σ is the standard deviation of the normal distribution of seed deaths over time.

Storing seeds hermetically at different levels of seed moisture content, *m* (% fresh weight basis) at the same temperature, *t* (°C) and at different temperatures with the same moisture content, Ellis and Roberts (1980a,b) found that there was a logarithmic relationship between σ and *m*, and a semi-logarithmic quadratic relationship between σ and *t*. For both variables, σ increases as the variable is reduced, resulting in an extension in the length of time that the seeds remain viable during storage. Thus, σ can be used as a measure of the relative longevity of a sample of seeds (time for viability to fall, e.g., from 97.7 to 84.1%, or from 84.1 to 50%). These relationships are described by the equation:

$$\begin{array}{l} \log\sigma =K_{E}-C_{W}\log m-C_{H}t-C_{Q}t^{2} \end{array}$$

where *K*_E_ is a species constant that is the theoretical value of log σ when the seeds of that species are stored at 1% moisture content and 0°C; *C*_W_ is a species constant that describes the effect of change in moisture content on log σ for that species; and *C*_H_ and *C*_Q_ are species constants that describe the effect of change in temperature on longevity and which usually take the same values for all species (Dickie et al., 1990). There are limits to the logarithmic relationship between σ and *m* (Ellis et al., 1989; Roberts and Ellis, 1989), but no evidenced limits to the quadratic relationship between σ and *t* over the range of temperatures for which there is data (Dickie et al., 1990). It is worth noting that this continuity in the relations across broad ranges of temperature and moisture content does not suggest a shift in response due to reaching either the glass transition temperature or the monolayer moisture content.

Another factor that influences the isotherm is pressure (Ludwig and Macdonald, 2005; Okos et al., 2019). This is relevant for seeds in a few different contexts. Firstly, seeds are often equilibrated (e.g., dried for storage) at ambient atmospheric pressure, sealed inside an air-tight container, such as aluminum foil pouches, and then transferred to another temperature for storage. This will change the pressure within the container and thence have an effect on *a*_w_. For example, if the seeds are stored in an air-tight container at a lower temperature than the equilibration temperature, the pressure will decrease, as will the eRH of the seeds. However, these pressure and hence eRH changes are very small, and probably insignificant in terms of moisture relations and seed longevity. Sending seeds to space however, which has been attracting attention in recent years (e.g., Musgrave, 2002; Nechitailo et al., 2005; Tepfer and Leach, 2017), may mean that they are exposed to much lower pressures, where eRH is very much reduced. Conversely, experimental storage of seeds in pressurized tanks with a high partial pressure of oxygen is being promoted as an alternative way of accelerating the aging process to get a better understanding of seed longevity, particularly at low moisture contents (Groot et al., 2012; Buijs et al., 2020). However, the elevated pressure will shift the isotherm to higher eRH (Okos et al., 2019) (Supplementary Table 2); hence, the aging process may not accurately reflect aging under very dry conditions.

## Isotherm Methodology: Determination and Modeling

There are a number of sources of information about how to determine an isotherm (e.g., Bell and Labuza, 2000). Perhaps the most common method is to place samples in different controlled humidity environments. Different humidities are often created using different saturated salt solutions, over which the sample is placed, within an air-tight container (e.g., Vertucci and Roos, 1993). Tabulated values of the RH produced by various salt solutions at different constant temperatures are readily available (see e.g., Rahman and Sablani, 2009) and the moisture content data are then plotted against these values. For some salts (e.g., LiNO_3_), temperature has a large effect on the RH produced, while for others (e.g., LiCl, NaCl), the RH produced is relatively stable across temperatures.

Alternatively, instead of using saturated salt solutions, electronically controlled environmental chambers can be used (see e.g., Lee et al., 2019), non-saturated solutions of LiCl (Hay et al., 2008) or conditioned (i.e., moistened) desiccant. Loggers that record RH (and often temperature) can be used to determine the RH in the environment if necessary. After equilibrium is expected to have been reached in the controlled environment (which may be followed by assessing changes in sample weight), the moisture content of the samples would be determined in the normal way, most commonly in the case of seeds, using the constant temperature oven method (ISTA, 2022). Alternatively, the water activity of the seed sample could be measured at regular intervals, using a water activity meter or hygrometer, to determine whether the seeds have equilibrated with the RH of the environment. Actual measurement of sample eRH may be done regardless, to get empirical data for isotherm modeling. Dynamic sorption instruments are also available; these instruments record the changing weight of a sample as the RH inside the sample chamber is changed (e.g., Rahman and Al-Belushi, 2006; Butler et al., 2009), although for heterogenous complex structures such as seeds, it might be difficult to program such an instrument to accurately predict the point of equilibrium, particularly at very low or very high relative humidities. In some of our own research, isotherms have also been constructed by measuring the eRH and then moisture content of seed samples taken from a drying bulk seed lot (Whitehouse et al., 2015). This can be efficient in terms of both determining an isotherm and following the drying process. It is recommended that the samples are hermetically sealed in a suitable container and stored for a few hours or days at the relevant temperature before eRH measurement, to ensure that the moisture is equilibrated throughout the seeds (i.e., within individual seeds and throughout the sample).

As well as there being different methods for collecting isotherm data, various equations (Supplementary Table 1, Figure 4) have been used to model the data and there are numerous papers in the food science literature where model fits for the different equations have been compared (e.g., Chen, 2003; Andrade et al., 2017; Zeymer et al., 2019; Mallek-Ayadi et al., 2020). This serves to emphasize that there should be caution in interpreting sorption isotherms, especially when parameters are given theoretical significance; the results will depend on the software used for analysis but, even more critically, on data quality and quantity (i.e., moisture range covered and number of points and replicates used for isotherm determination). Indeed, in our review of the literature, some isotherm equations were fitted to as few as four data points (Supplementary Table 1). This is particularly problematic if we want to infer something from the fitted relationship. In the seed literature, a more acceptable RH range and number of observations have tended to be used to plot isotherms.

In terms of theoretical models, the Brunauer-Emmett-Teller (BET; Brunauer et al., 1938) and related Gugenheim-Anderson-de Boer (GAB) equations have been widely applied in food science (Van den Berg, 1985), with the BET equation better describing the properties of water at high moisture content (Rahman and Al-Belushi, 2006). They both include a monolayer value, *M*_m_, which is determined as part of the fitting process and which is the theoretical amount of water adsorbed on surfaces and in capillaries; at higher moisture contents, water can behave as a solvent (Bell and Labuza, 2000). This is why *M*_m_ is considered the point where stability is maximized (Figure 2). However, these equations are incorrect from a theoretical point of view caused by the absence of Henry's law limit; as the RH tends to zero, the moisture content tends to finite values, whereas it should tend to zero (Labuza and Altunakar, 2020). Furthermore, these theoretical equations are often out-performed by empirical or, if temperature is included, semi-empirical equations, including for example, Caurie, Chung-Pfost, Copace, Halsey, Henderson, Oswin, Peleg and Smith, and their respective modifications (Chen, 2003; Andrade et al., 2017; Zeymer et al., 2019; Mallek-Ayadi et al., 2020) (Figure 4, Supplementary Table 1, Supplementary Figure 1).

The D'Arcy-Watt equation (D'Arcy and Watt, 1970) has been used in a number of seed science papers (e.g., Vertucci and Leopold, 1987; Welbaum and Bradford, 1989; Sun et al., 1997; Vashisth and Nagarajan, 2009; Socorro García et al., 2010). This equation reflects a theoretical process of moisture sorption, whereby water is primarily adsorbed to strong binding sites at low moisture levels; at mid-water contents, more water is weakly bound and this increases as eRH increases; and at high moisture levels, there is multi-molecular water (Figure 4C). The original D'Arcy-Watt equation is not widely applied in the food literature, although more recently, a “generalized” form of the equation, which more realistically describes the binding of successive water molecules as water content increases (Furmaniak et al., 2005), has been used to model the isotherm data of various food products (Furmaniak et al., 2007; Vasile et al., 2020). In this Generalized D'Arcy-Watt (GDW) model, all unrealistic assumptions of the BET concept are omitted. The GDW assumes the existence of primary (Langmuir-type) adsorption sites that can adsorb only one water molecule. Those water molecules can then be the secondary adsorption sites for other water molecules. Two differences from the original model are that not all water molecules adsorbed on primary sites can be secondary sites for other water molecules, and it provides the possibility that one water molecule attached to a primary site can create more than one secondary adsorption site (Furmaniak et al., 2007).

Although not an isotherm equation *per se*, Cromarty's equation also describes the relationship between seed moisture content and RH, depending on the temperature and the oil content of the seeds (Cromarty et al., 1982; Figure 4D). It was suggested that it could be used to determine the drying conditions required for seeds to reach the target moisture content for long-term genebank storage, though it is more common now, for seeds intended for genebank storage, to all be dried under the same conditions regardless of species, and actual moisture content is rarely determined (FAO, 2014). Cromarty's equation requires knowledge of seed oil content, but such data is available for many species. It should also only be used for seeds equilibrated to between 10 and 70% RH and 0–40°C for starchy seeds and 15–25°C for oily seeds (Cromarty et al., 1982).

## Isotherms in Seed Science: Informing Longevity, Dormancy and Germinative Metabolism

As illustrated in Figures 1, 2, moisture sorption isotherms can be used to suggest the type of chemical reactions occurring in seeds. In fact, arguably, it is only relevant to know the eRH of the seeds and not even necessary to determine an isotherm or moisture content (percentage or proportion of fresh or dry weight). For example, germination processes cannot start until there is sufficient multi-molecular water to support respiration and the re-establishment of metabolism (Figure 1). Vertucci and Leopold (1984) detected some oxygen depletion in region II of the moisture adsorption isotherm, but rapid increase associated with respiration only occurred when the seeds had reached moisture contents in equilibrium with >85% relative humidity. This is similar to the upper moisture limit to the negative relationship between seed longevity and moisture content (Figure 2) described by the viability equations (Ellis and Roberts, 1980a,b; Box 1): 90% in niger and lettuce, and 85.4–88.8% for tef (Zewdie and Ellis, 1991). This limit, above which increasing moisture content did not have the same negative effect on longevity was attributed to the onset of repair processes (Villiers, 1974), which is now recognized as an important element of the seed longevity response (Waterworth et al., 2015, 2019). Macromolecular repair also explains the potentially longer than expected “persistence” of seeds in the soil seed bank, where they may experience periods where they are, in effect, in region III of the isotherm and at other times, in region II (Figure 1), a phenomenon known as wet-dry cycling (Long et al., 2011). Repair processes do, however, require oxygen to be present; under hermetic storage conditions or water-logged soil, there may not be sufficient oxygen to support prolonged activation of such metabolism, and hence longevity will be negatively impacted if seeds are at such a high moisture content for long periods (Figure 2).

Orthodox seeds are, however, normally stored at a moisture content that is within region II of the isotherm (Figures 1, 2) and often, in particular in long-term genebank storage, hermetically (FAO, 2014). Thus, oxidative and auto-oxidative aging reactions, will, due to lower levels of oxygen, be reduced. According to the food stability diagram (Figure 2), while oxidative reactions reduce as the eRH decreases to about 30%, at lower eRH, auto-oxidation increases. Thus, if seeds are stored at low moisture content in an open system, we might expect aging rates to increase (as reported by Vertucci and Roos, 1993) due to these auto-oxidative reactions. Under this open storage, the values of the optimal moisture contents for seed storage correspond to the values determined for saturation of strong binding sites (BET monolayer value), both for pea cotyledons and *Typha latifolia* pollen (Buitink et al., 1998; Walters, 1998; Ballesteros and Walters, 2011). Although these values are situated below the *T*_g_, they do run in parallel to the *T*_g_ curve. This increase in instability at lower moisture contents is largely negated when seeds are stored hermetically and this explains the long-term maintenance of viability of seeds stored hermetically under ultra-dry conditions (e.g., Pérez-García et al., 2009), for which, according to FAO/IPGRI (1994), seeds may be dried using silica gel or to equilibrium with 10–12% RH at 20°C. This recommendation came from cross-referencing estimates of the low moisture content limit to the applicability of the viability equations to sorption isotherms (Ellis et al., 1989, 1992), although it was subsequently accepted that the RH where the low critical moisture content occurred increased as the storage temperature decreased (e.g., from 10.4–10.8 at 65°C to 13.1–13.5 at 39.9°C in the case of *Trifolium pratense* and *Medicago sativa*; Ellis and Hong, 2006). Because then, there is a clear RH range over which the viability equations are applicable, and since seeds are normally stored within this range (i.e., “air-dry”), for studies of comparative longevity of seeds of different taxa it makes sense that seeds are also stored at an equilibrium RH within this same range (Hay et al., 2019); more precisely, an RH of 60% has been recommended (Newton et al., 2014; Hay et al., 2022) as this RH is sufficiently high for the aging process to be accelerated such that viability loss can be followed over days–months, depending on the storage temperature, but not so high that enzymic reactions occur at a significant rate. Indeed, a number of papers have reported different relative rates of aging of seed lots at ≥75% RH (controlled deterioration conditions) *cf*. storage at lower RH (Schwember and Bradford, 2010; Hay et al., 2019).

Although most seed storage studies have controlled and presented the moisture content of the seeds during storage, Roberts and Ellis (1989) considered both the limits of applicability of the viability equations in terms of eRH (see above) and the actual relationship between seed longevity and eRH. They found that, unlike the logarithmic relationship between σ and moisture content (Box 1), the relationship between σ and eRH was semi-logarithmic. However, as pointed out by Hay and Timple (2016), this is somewhat unexpected since, in region II of the isotherm where the viability equations apply, the relationship between moisture content and eRH is approximately linear (Figures 1, 2). In fact, Hay and Timple (2016) concluded that there was a logarithmic relationship between longevity and eRH. Nonetheless, Roberts and Ellis (1989) also concluded that the slope of the semi-logarithmic relationship between longevity and eRH was the same, at least for two very contrasting species: lettuce and barley (Figure 2). That is, while the quantitative effect of change in moisture content on seed longevity varies between species, the effect of change in eRH was the same. However, there was also an impact on the temperature effect; when moisture content was used, the effect of changes in temperature on σ were constant across species (Dickie et al., 1990), but, when eRH was used, the effect of changes in temperature on σ varied across species. Presumably then, since the effect of change in moisture content on longevity differs between species and depends on temperature, it could be correlated with the slope of the isotherm within region II, or accordingly, some parameter(s) of a suitable isotherm equation.

Seed aging is not the only process that occurs in seeds when they are in an air-dry state (i.e., region II of the isotherm). In *Bromus tectorum* L. seeds, dormancy release (“dry after-ripening”) occurred when the seeds were at water potentials between −350 and −80 MPa (Bair et al., 2006), equivalent to eRH values of approximately 8 and 75%, respectively. Similarly, dormancy release in *Lolium rigidum* Gaud. seeds occurred when moisture content was within region II of the isotherm (Steadman et al., 2003). The effect of various combinations of temperature (10–30°C) and relative humidity (1–85%) on dormancy alleviation was investigated in sunflower and Arabidopsis seeds during dry after-ripening (Bazin et al., 2011; Basbouss-Serhal et al., 2016). In both species, the rate of dormancy alleviation depended on both temperature and embryo moisture content. For sunflower, dormancy release was faster at 15°C than at higher temperatures when the embryo moisture content was <10% (dry weight basis; approximately 75 or 85% eRH for embryonic axes and cotyledons, respectively, at 15°C) (Bazin et al., 2011). Thermodynamic analyses showed that water-binding properties changed during dormancy alleviation toward less bound water being available. It was concluded that this change controls the nature of the reactions involved in the transition from the dormant to a non-dormant state (Bazin et al., 2011). A similar change in the kinetics of dormancy release was found for Arabidopsis seeds, for which the rate of dormancy release was fastest at about 50% RH (Basbouss-Serhal et al., 2016).

The above are examples of what can be inferred from knowing eRH rather than moisture content, however there are also examples where isotherms have been determined with an exploratory perspective, to see whether differences in isotherms are correlated with differences in physiological response. For example, Welbaum and Bradford (1989) associated the desiccation intolerance of very immature muskmelon seeds with a lack of strongly bound water based on fitting the D'Arcy-Watt equation (Figure 4C) to isotherm data. Isotherms have also been determined for non-orthodox seeds, in order to express the degree of desiccation tolerance in terms of both moisture content and eRH (and sometimes water potential) (Probert and Longley, 1989; Dussert et al., 1999; Eira et al., 1999). In relation to orthodox seeds, Sun et al. (1997) attributed a reduction in the longevity of primed mung bean seeds to a reduction in the amount of water in strong and multi-molecular binding sites and an increase in the amount of weakly-bound water, as determined by fitting the D'Arcy-Watt equation to isotherm data for primed and non-primed seeds. Vashisth and Nagarajan (2009) also reported shifts in water binding properties in magnetically primed maize seeds. Vertucci and Roos (1993) used isotherms to determine the optimum storage moisture content of pea, soya bean and peanut seeds over a wide temperature range; Socorro García et al. (2010) took a similar thermodynamic approach to relate sorption properties with long-term storage; Tangney et al. (2022) determined isotherms to assess risk of fire-induced death of buried seeds.

## Perspectives

In terms of understanding the metabolic activity of orthodox seeds, eRH is a simpler, non-destructive method of assessing moisture status that can be interpreted on the basis of isotherm region and for example, relative rate of viability loss or whether there is active germinative metabolism (Figures 1, 2), even without determining an actual isotherm. For example, if seeds have an eRH around 25%, the rate of aging would be relatively slow; if the eRH is around 70%, the rate of aging would be relatively fast; and if the eRH is around 98%, if oxygen is available, the seeds would be “preparing” for germination (Figure 2). Thus, in general in seed science, more effort could be made to determining eRH rather than moisture content and there are suitable, reliable laboratory instruments for this.

Isotherms have normally been determined over what could be considered a limited range of more “ambient” temperatures (e.g., 5–50°C). Most notably, they are not normally determined at sub-zero temperatures. In contrast, for long-term conservation, seeds are routinely stored at −20°C. Vertucci and Roos (1993) estimated isotherms at sub-zero temperatures using linear van't Hoff analysis of isotherm data collected at 5–50°C and extrapolating −150°C. They used these extrapolations to estimate the optimum seed moisture content for genebank storage. However, the isotherms that were experimentally determined by Vertucci and Roos (1993) are quite distinct in that the isotherms do not converge until the equilibration RH is very low [compare for example, adsorption isotherms for pea seeds in Vertucci and Roos (1993) and Chen (2003); see also, e.g., Maleki Majd et al. (2013), Mousa et al. (2014), Talla (2014)]. Furthermore, it has recently been recognized that non-linear van't Hoff analysis is less erroneous than linear analysis (Lima et al., 2020). Thus, there is a need for seed scientists, particularly those involved in seed conservation, to determine desorption isotherms at a range of temperatures, evaluate different isotherm equations to model the data and reconsider how to extrapolate to low temperatures to improve our understanding of seed moisture relations and hence longevity at genebank temperatures.

There are considerations that need to be taken into account in moving toward determining isotherms and using eRH more systematically. Hysteresis (Figure 2) is one of these; while in theory, eRH should be informing relative rate of aging, it has been found that desorping and adsorping seeds of rice at the same moisture content rather than the same eRH had the same rate of probit viability loss (Bello et al., 2011; Hay and Timple, 2016), thus until more is understood about the hysteresis loop and its effect on aging reactions, it seems it is important to also be knowledgeable of the moisture history of the seeds being used. It may be better to dry the seeds to a low moisture content, before starting any experiments where control of moisture content is required. How low, however, is unclear, and may vary between species. Whether the change in the shape of the isotherm due to the physiological status of the seeds (for example, dormant vs. non-dormant or primed vs. non-primed) is related to a time-dependent change of the hysteresis effect or due to a change in water properties that in turn can explain the differential seed behavior merits further attention. None of the equations mentioned in this paper took into account the hysteresis effect; many are used to separately describe either ad- or desorption data. It would be helpful for example, if Cromarty's equation could be modified with an additional parameter to account for the shift in the moisture content-eRH relationship when seeds are adsorbing moisture rather than just desorbing. Then, a new prediction of moisture content for adsorbing seeds could be inserted into the viability equations to predict loss of viability during storage. Cromarty's equation is the only one considered here that describes the moisture content-eRH relationship based on composition, specifically oil content, with the “isotherm” shifting to lower moisture contents the higher the oil content (Figure 4D). It might be helpful if other equations might be similarly parametrized to take into account composition, though the theoretical equations suggest that this would perhaps be over-simplification of the properties of water binding.

It should also be noted that there is perhaps less value in measuring the eRH rather than moisture content for recalcitrant seeds below region III of the moisture isotherm (Figure 1). Recalcitrant seeds are generally dispersed with a high moisture content and are metabolically active (Pritchard et al., 2004; Wyse and Dickie, 2017). Drying recalcitrant seeds below region III of the isotherm results in loss of viability, a consequence described as “desiccation intolerance”. Indeed, this was the basis of an alternative classification of seed storage behavior, with types I, II and III corresponding to orthodox, intermediate and recalcitrant seeds, respectively (Pritchard, 2004). Seed eRH is nonetheless still relevant in relation to cryopreservation protocols of non-orthodox species. For example, Hor et al. (2005) found that for the intermediate oily seeds of *Citrus* species, drying seeds of the different species at 75–80% RH (i.e., close to the boundary between regions II and III) consistently resulted in the removal of freezable water, and resulted in maximum survival following cryostorage, though the moisture content varied among the different species.

Seed moisture content as a test has been validated by ISTA, meaning that moisture content results should be reproducible among laboratories accredited to perform the moisture content test. Measurement of seed eRH has not yet been validated across laboratories as an alternative to determining seed moisture content, although efforts are being made in that direction. Where it is only possible to determine moisture content, published isotherms might be used to estimate eRH (Supplementary Table 1, Supplementary Figure 1), though the estimate might be subjected to error due to differences in the method to determine moisture content as well as cultivar and seed lot variation in water sorption properties, with the latter being due perhaps to drying and moisture history, and/or other treatments. In relation to coating treatments, a film coating, depending on its composition and thickness, may not alter the moisture content-eRH relationship to the extent that measuring eRH to understand the moisture status of the coated seed is problematic. In contrast, the eRH measured for encrusted or pelleted seeds (Pedrini et al., 2018) may not so accurately reflect that of the seed inside, again depending on the nature and moisture sorption properties of the ingredients used.

In writing this paper, we have compiled sources of seed isotherm data from the food science and seed science literature (Supplementary Table 1, Supplementary Figure 1). This is no doubt not exhaustive, but perhaps a starting point for considering isotherms as a seed trait that is useful for further analyses and/or to inform the design of storage, dormancy-alleviation, and other experiments. In terms of gaining a more complete understanding of, and optimizing, the seed drying process and storage requirements, we encourage more holistic studies in which isotherms and glass transition temperatures at different seed moisture contents are determined alongside seed storage experiments for the same seed lot. This may be particularly beneficial for genebanks who aspire to maintain the viability of seeds for many decades.

## Data Availability Statement

The original contributions presented in the study are included in the article/Supplementary Material, further inquiries can be directed to the corresponding author.

## Author Contributions

SR and FH conceived the paper. All authors contributed to the writing and editing of the manuscript and approved the submitted version.

## Funding

The writing of this review was supported by an AUFF Starting Grant to FRH (AUFF-E-2019-7-27).

## Conflict of Interest

The authors declare that the research was conducted in the absence of any commercial or financial relationships that could be construed as a potential conflict of interest.

## Publisher's Note

All claims expressed in this article are solely those of the authors and do not necessarily represent those of their affiliated organizations, or those of the publisher, the editors and the reviewers. Any product that may be evaluated in this article, or claim that may be made by its manufacturer, is not guaranteed or endorsed by the publisher.
