# Supplementary material for: Seed Moisture Isotherms, Sorption Models, and Longevity
Source: Front Plant Sci. 2022 Jun 2;13:891913. doi: 10.3389/fpls.2022.891913 (PMC9201756; doi:10.3389/fpls.2022.891913)
Supplement: Supplementary file 3 [file Presentation_1.PPTX]

## Slide 1
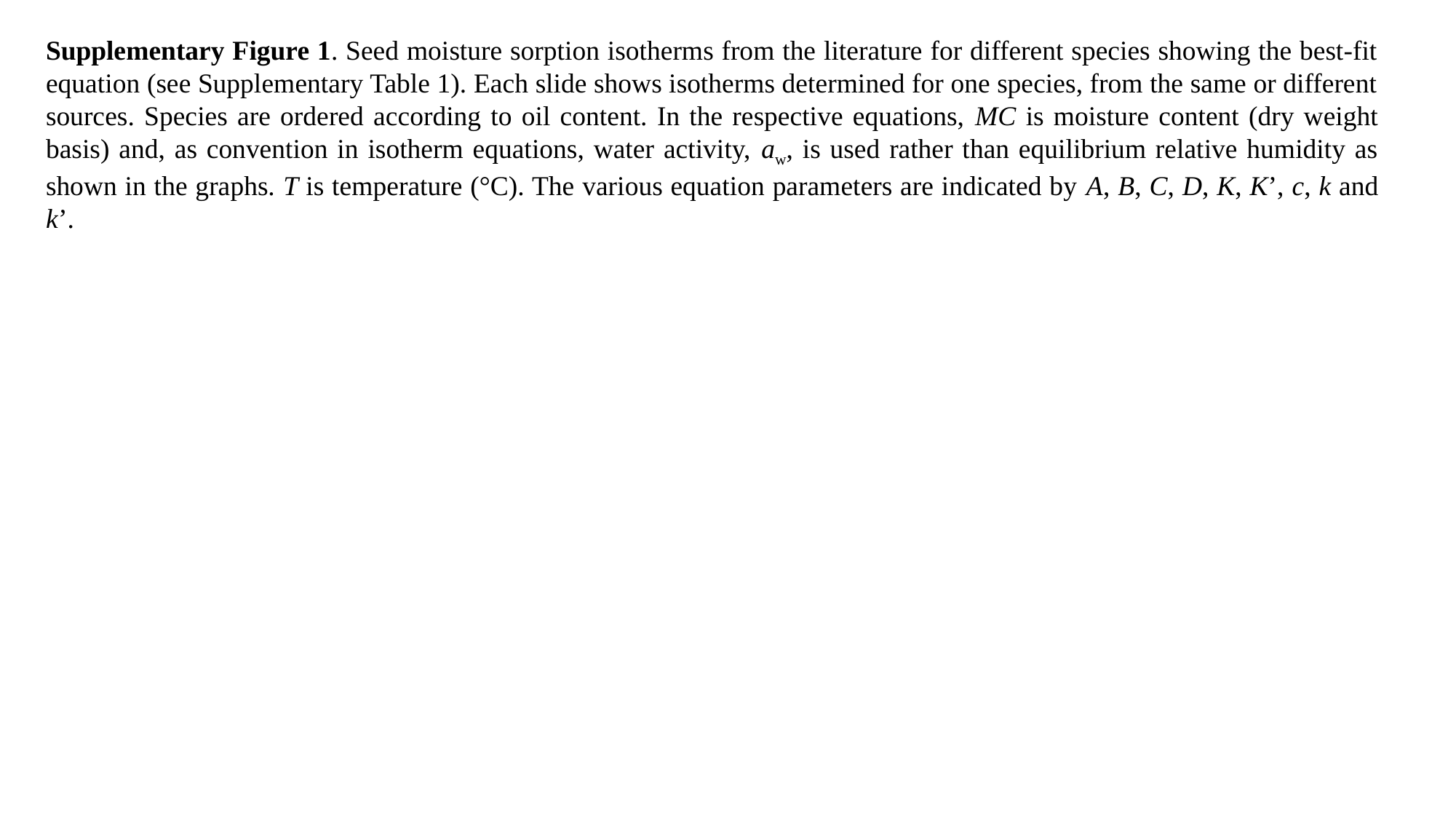

Supplementary Figure 1. Seed moisture sorption isotherms from the literature for different species showing the best-fit equation (see Supplementary Table 1). Each slide shows isotherms determined for one species, from the same or different sources. Species are ordered according to oil content. In the respective equations, MC is moisture content (dry weight basis) and, as convention in isotherm equations, water activity, aw, is used rather than equilibrium relative humidity as shown in the graphs. T is temperature (°C). The various equation parameters are indicated by A, B, C, D, K, K’, c, k and k’.

## Slide 2
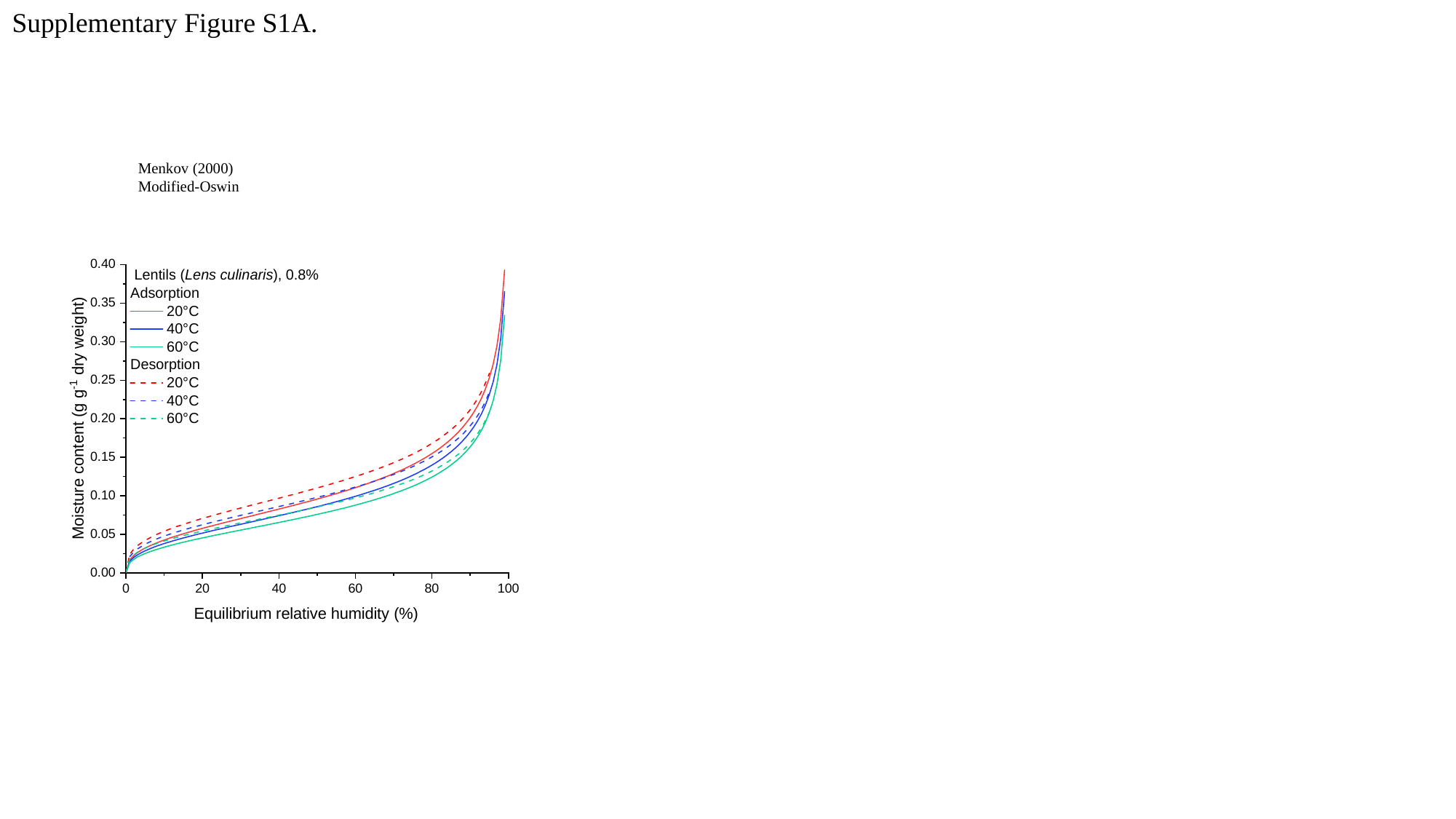

Supplementary Figure S1A.
Equilibrium relative humidity (%)

## Slide 3
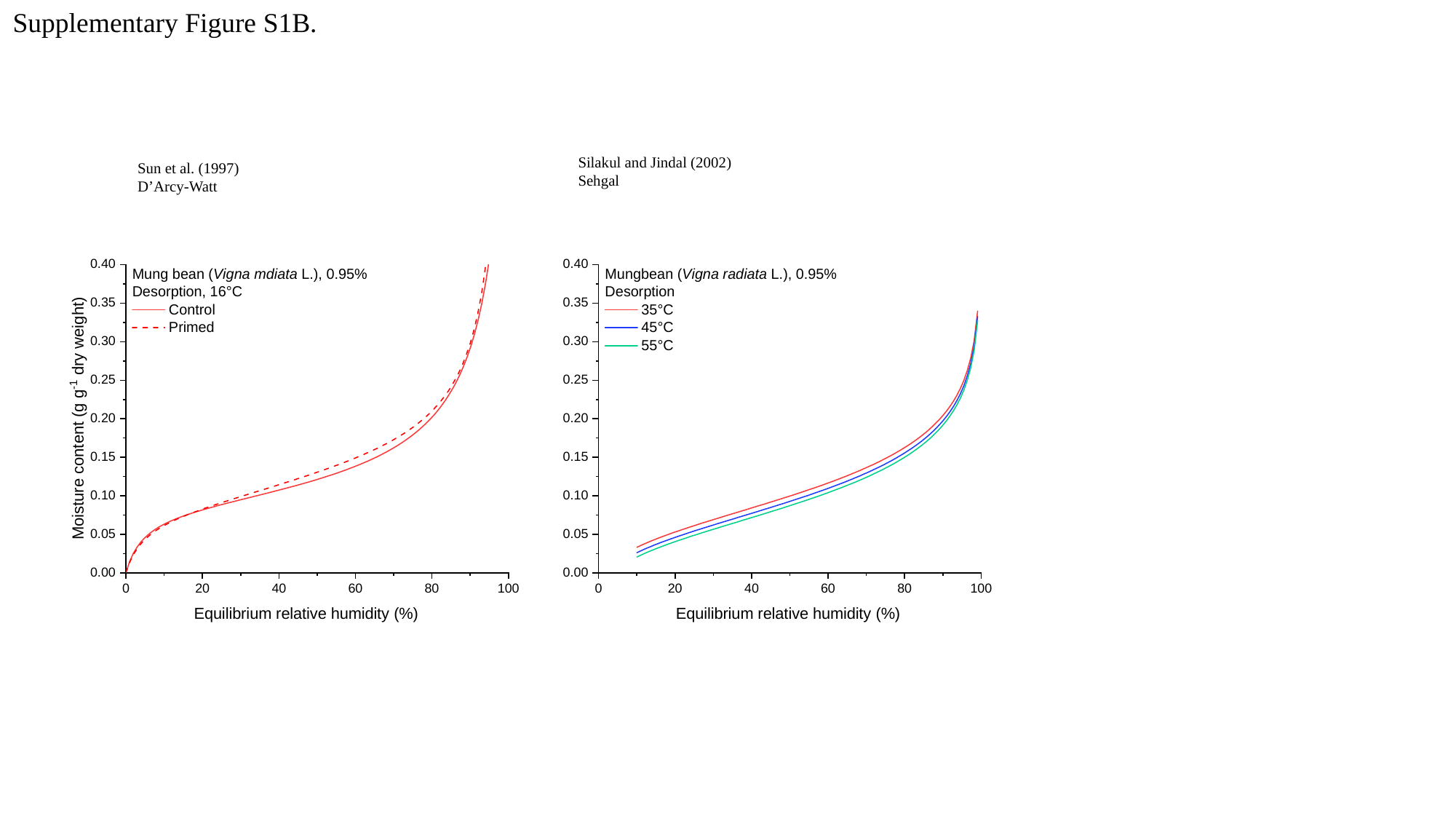

Supplementary Figure S1B.
Equilibrium relative humidity (%)
Equilibrium relative humidity (%)

## Slide 4
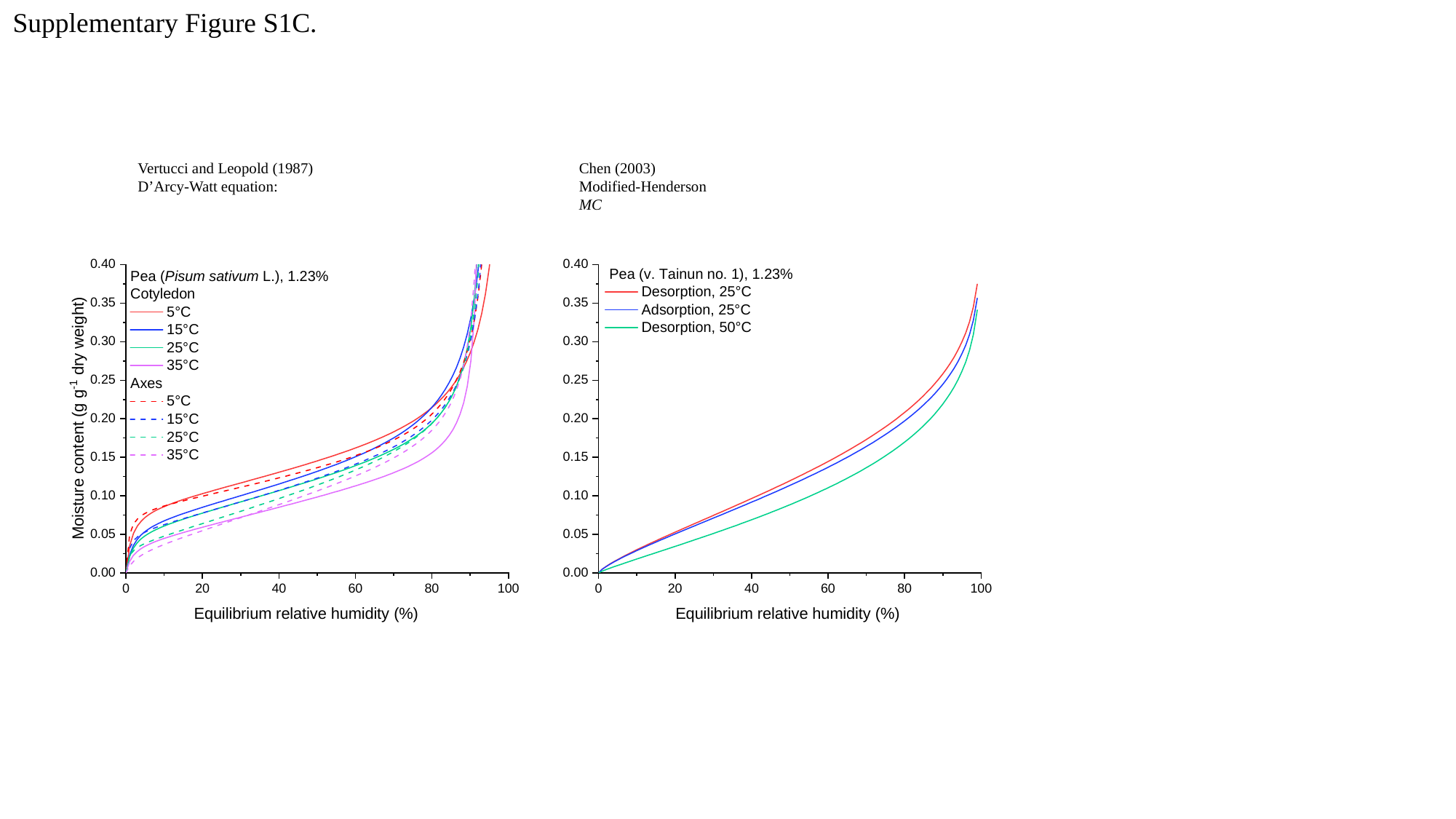

Supplementary Figure S1C.
Equilibrium relative humidity (%)
Equilibrium relative humidity (%)

## Slide 5
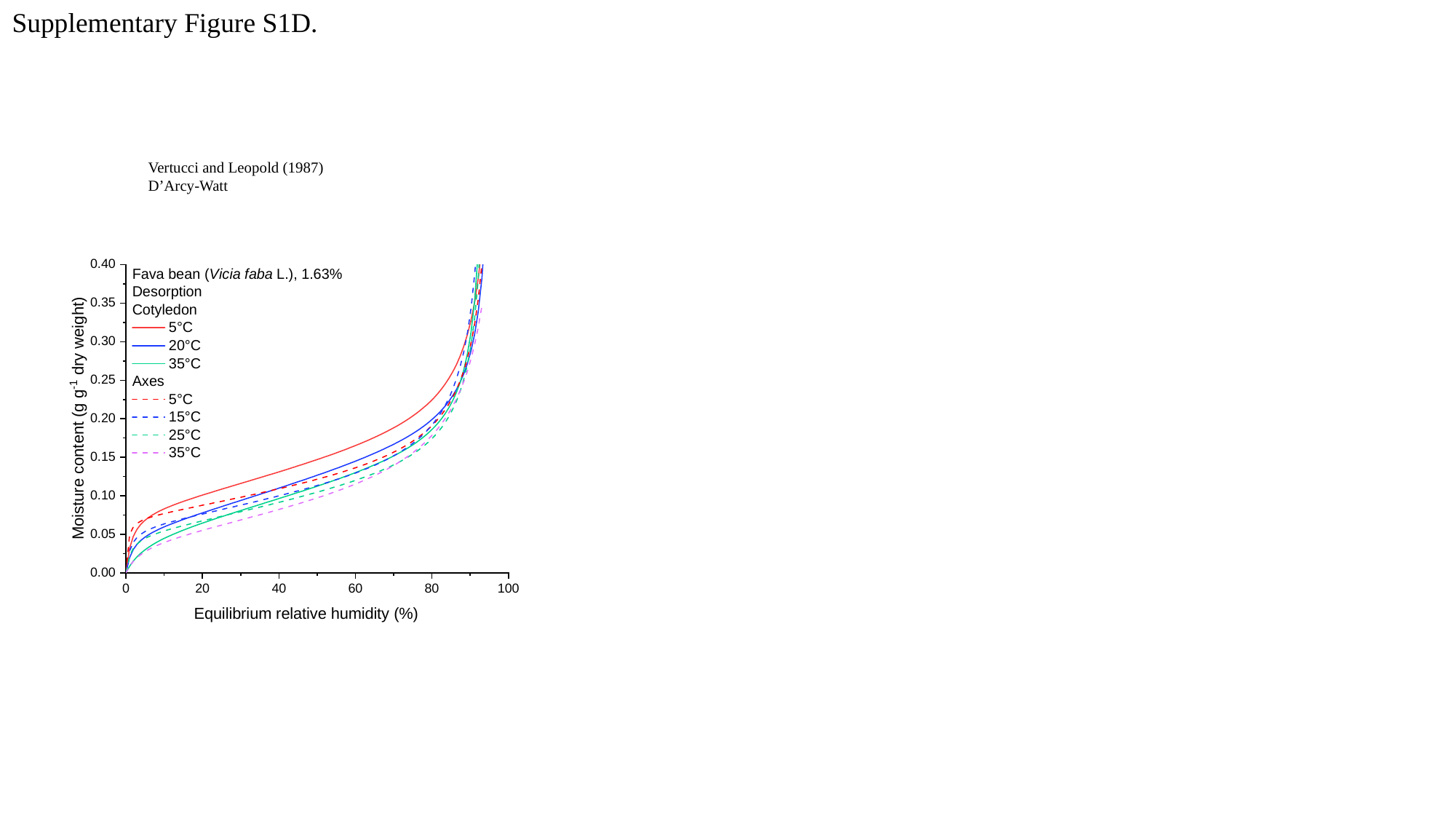

Supplementary Figure S1D.
Equilibrium relative humidity (%)

## Slide 6
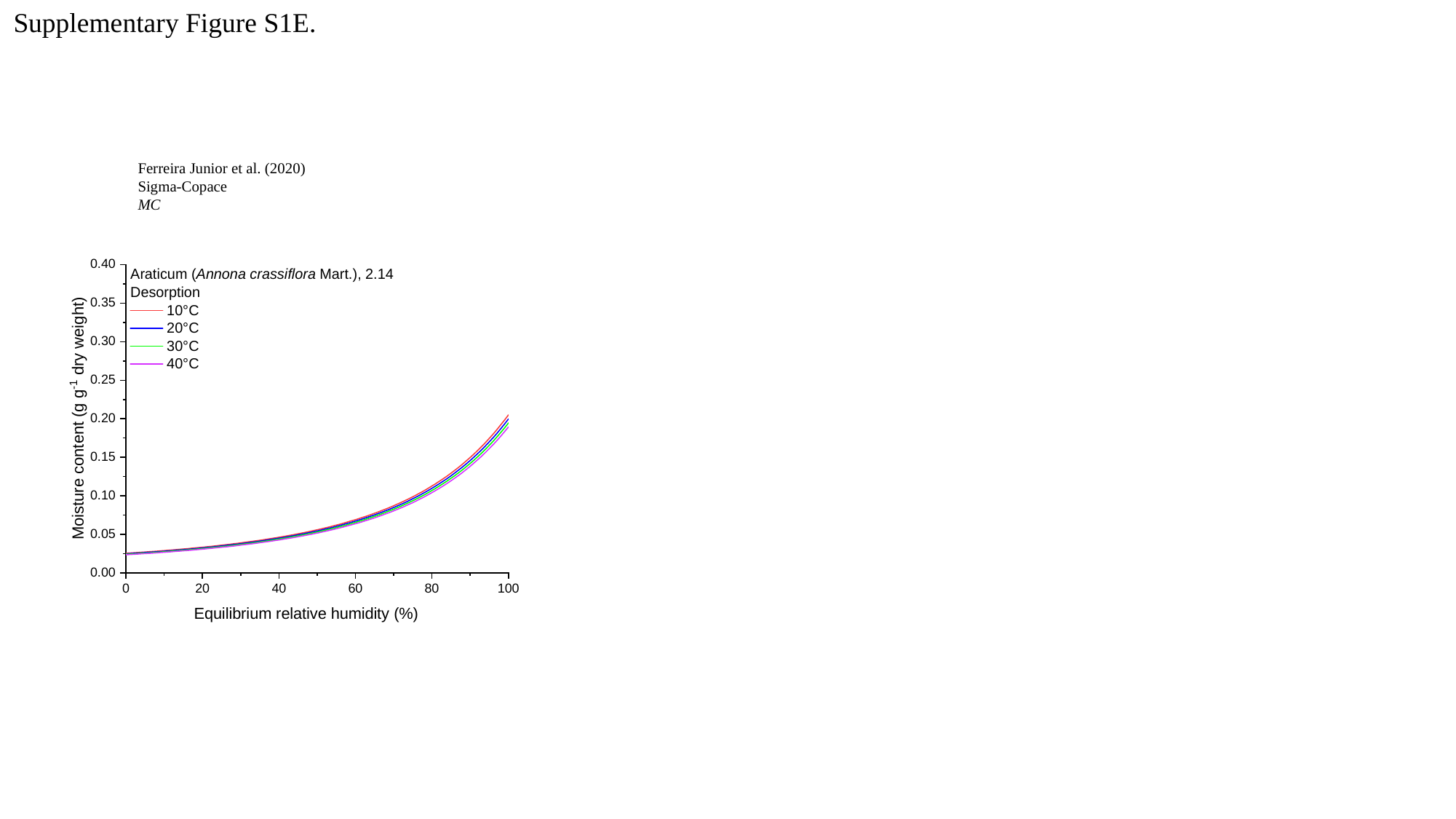

Supplementary Figure S1E.
Equilibrium relative humidity (%)

## Slide 7
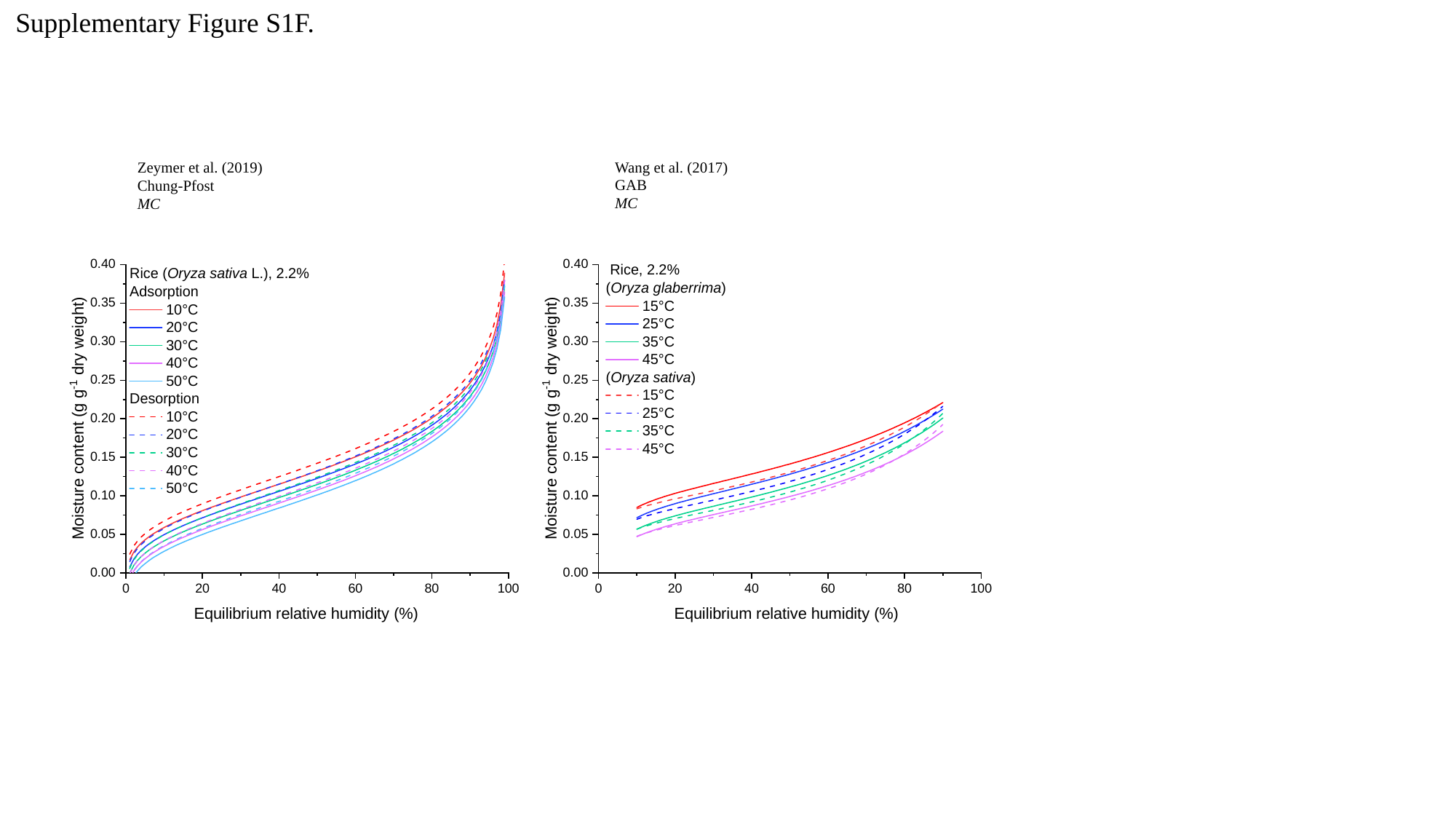

Supplementary Figure S1F.
Equilibrium relative humidity (%)
Equilibrium relative humidity (%)

## Slide 8
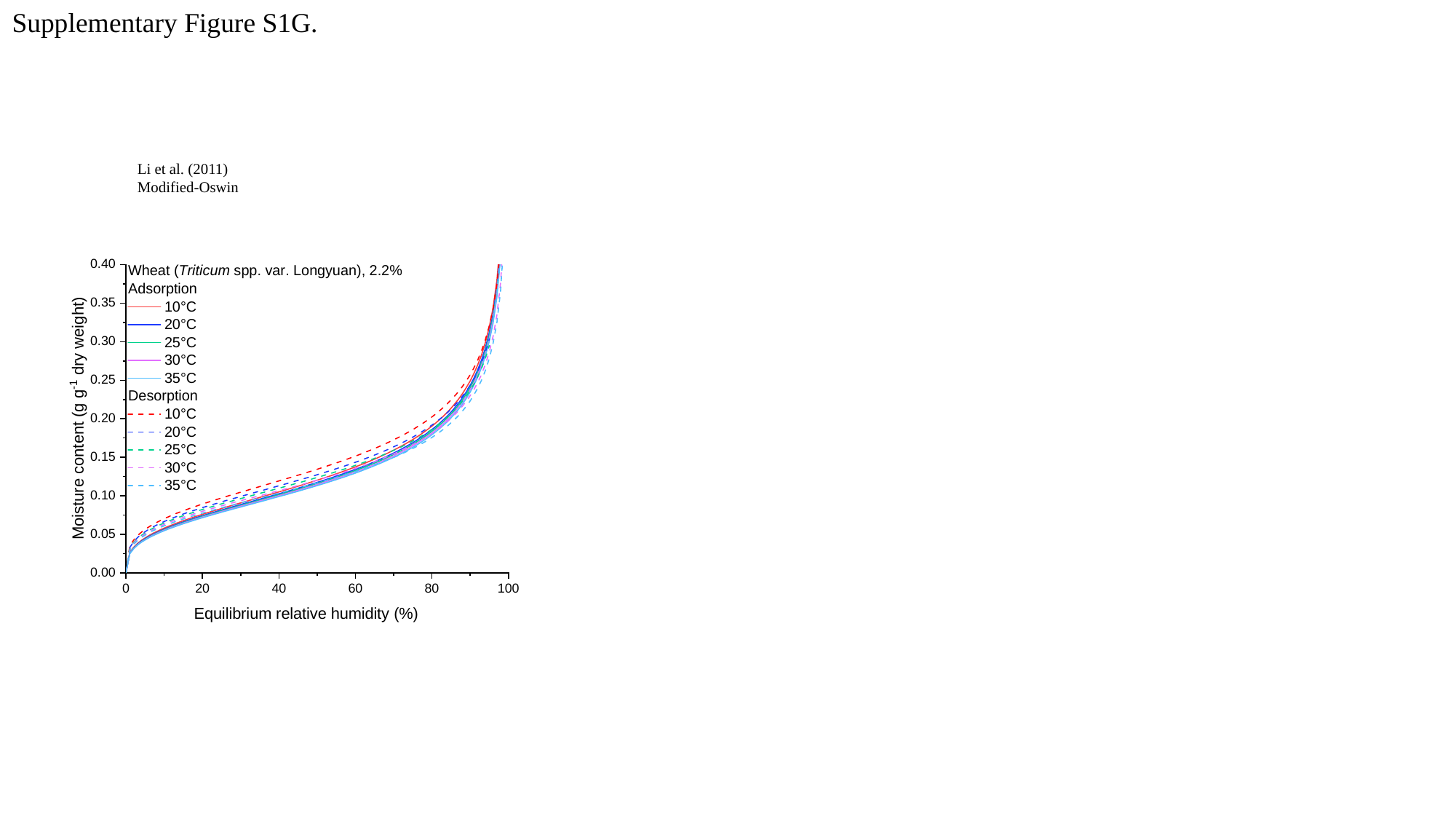

Supplementary Figure S1G.
Equilibrium relative humidity (%)

## Slide 9
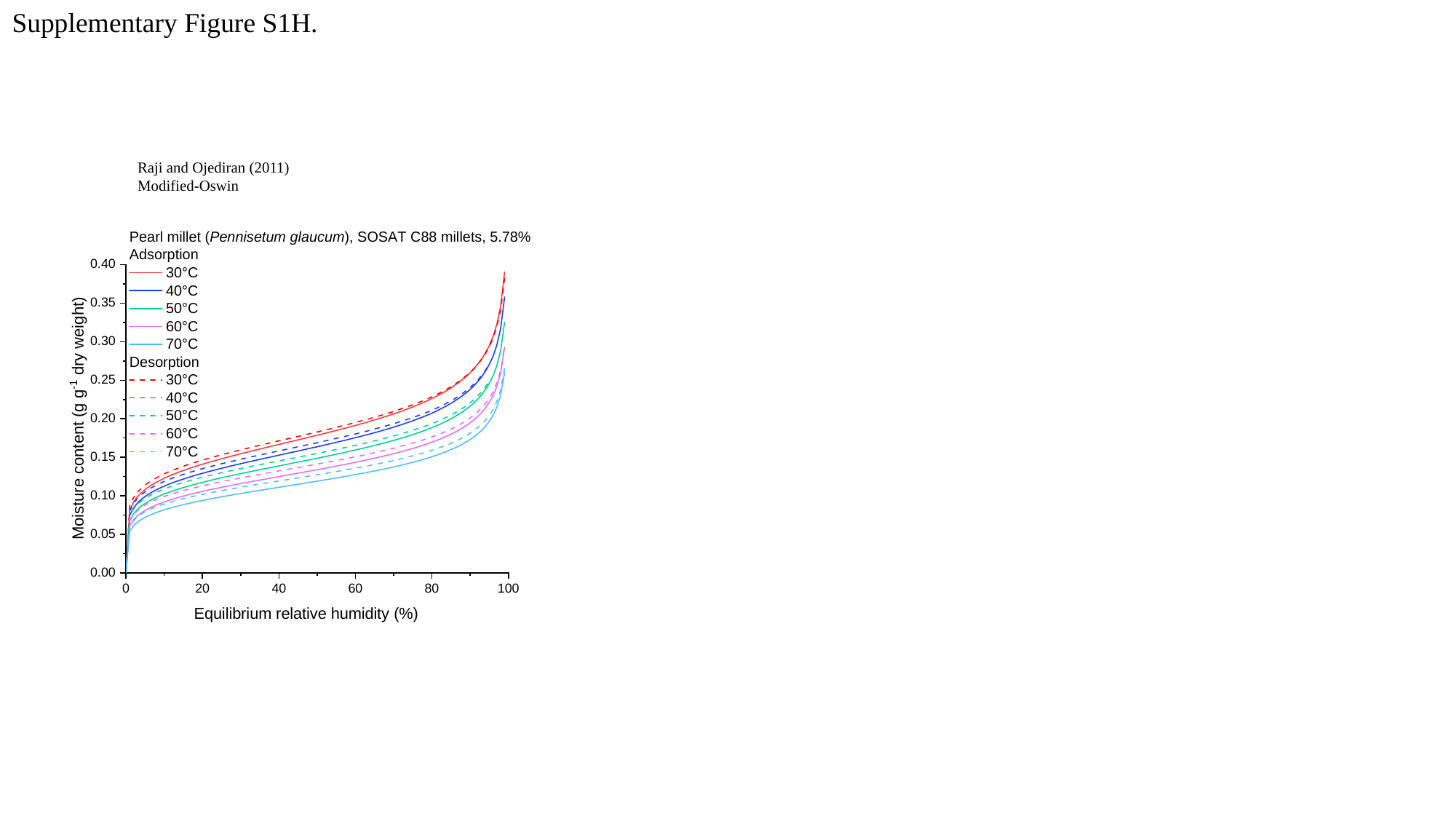

Supplementary Figure S1H.
Equilibrium relative humidity (%)

## Slide 10
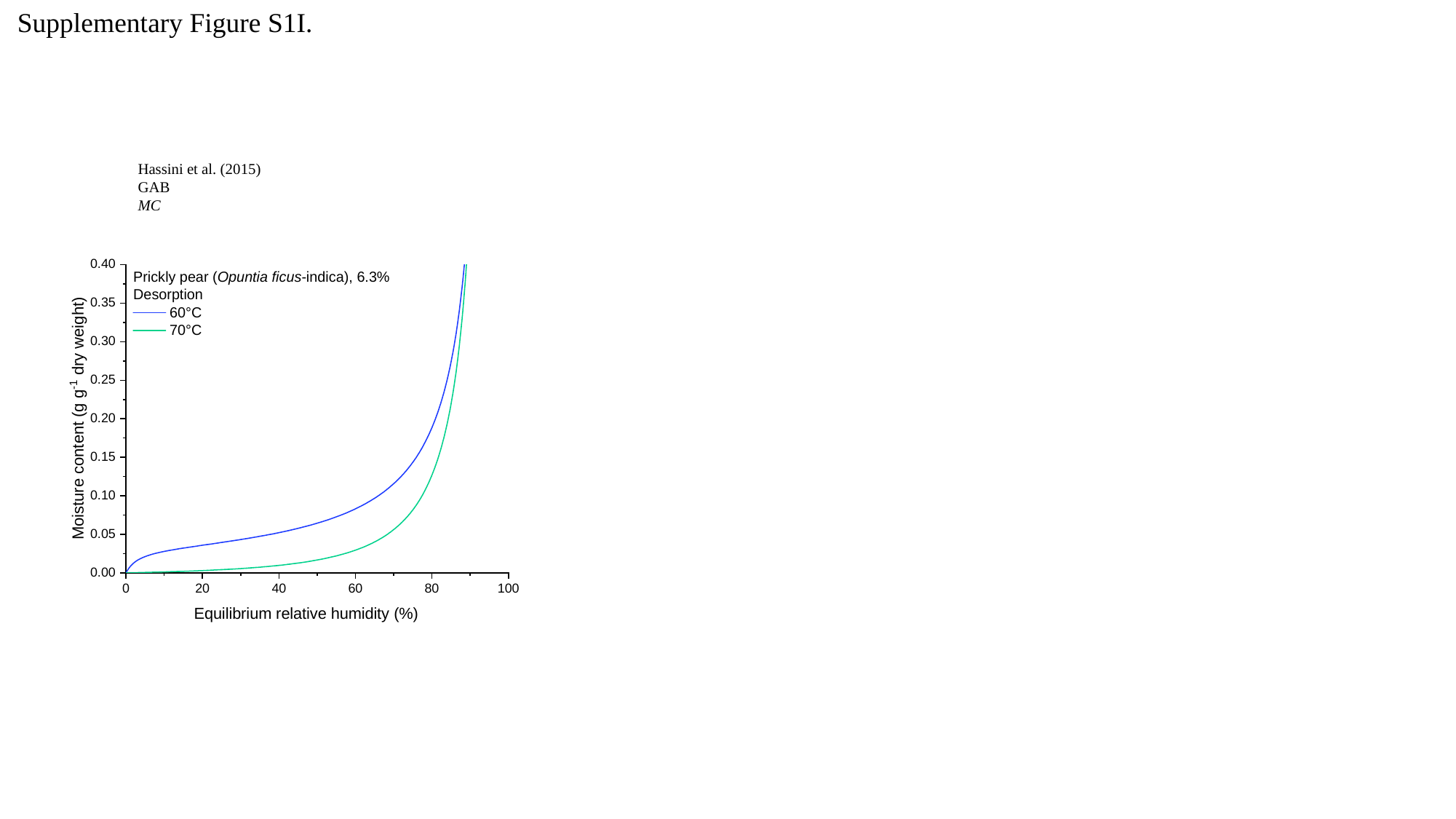

Supplementary Figure S1I.
Equilibrium relative humidity (%)

## Slide 11
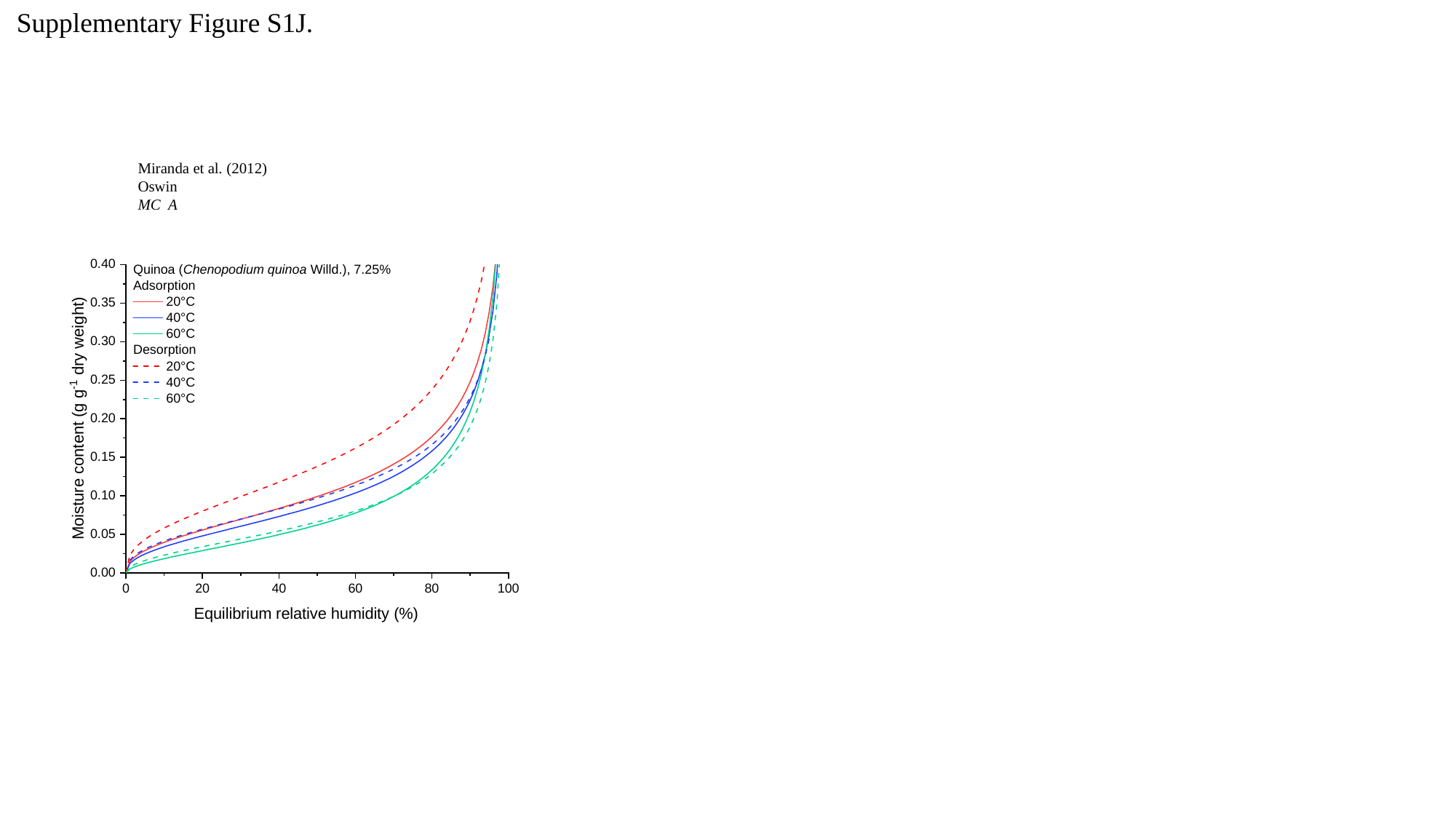

Supplementary Figure S1J.
Equilibrium relative humidity (%)

## Slide 12
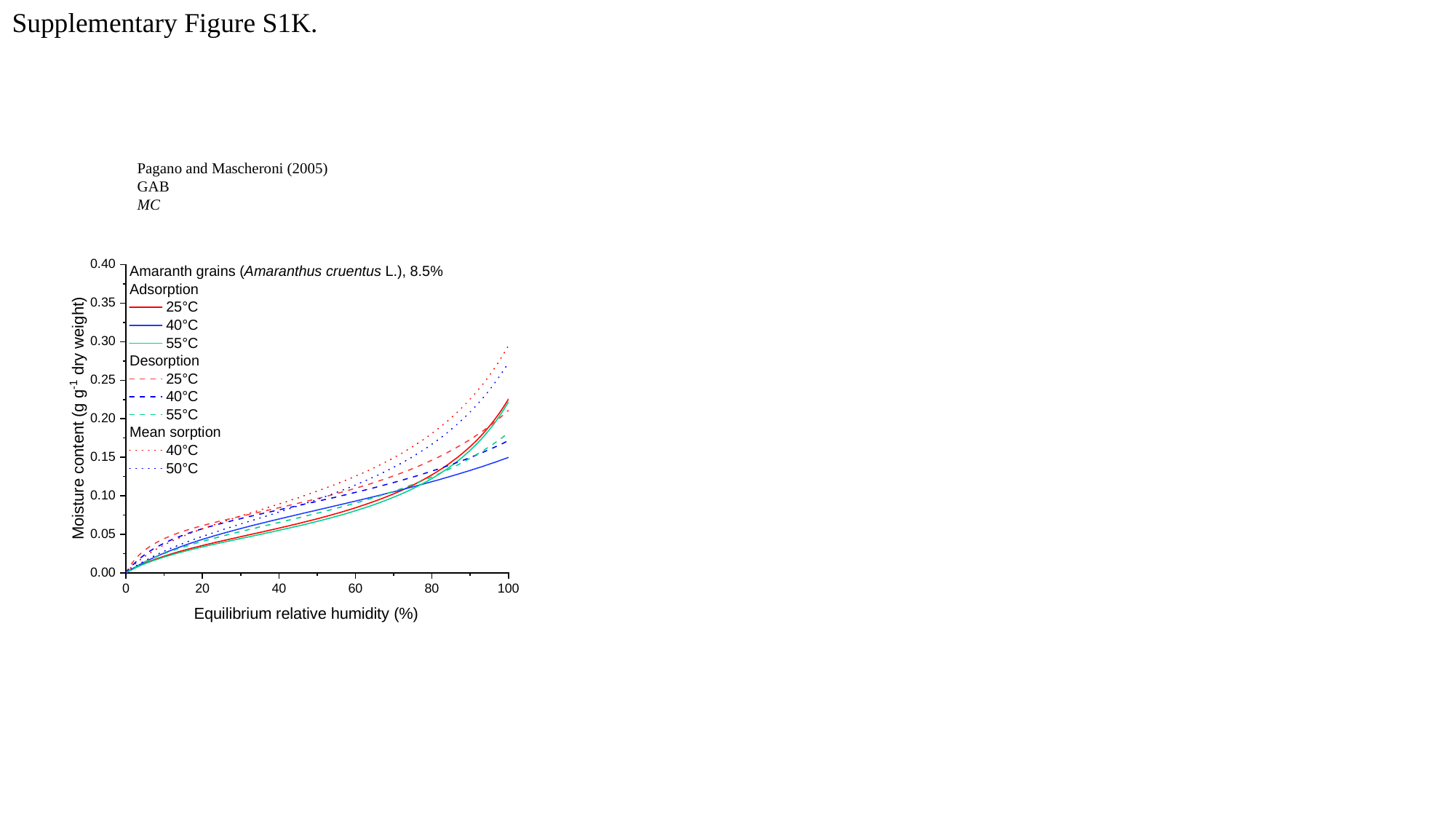

Supplementary Figure S1K.
Equilibrium relative humidity (%)

## Slide 13
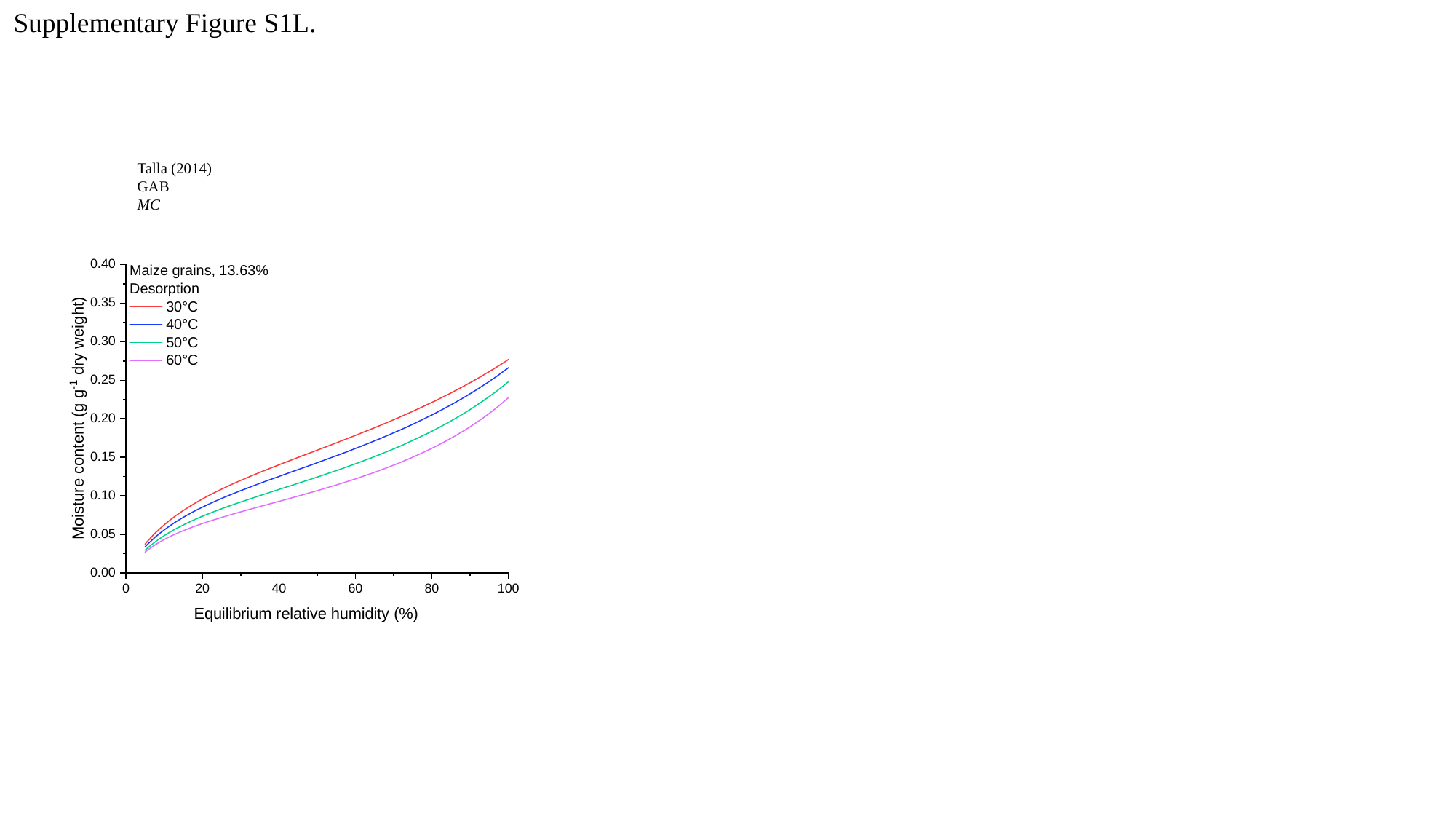

Supplementary Figure S1L.
Equilibrium relative humidity (%)

## Slide 14
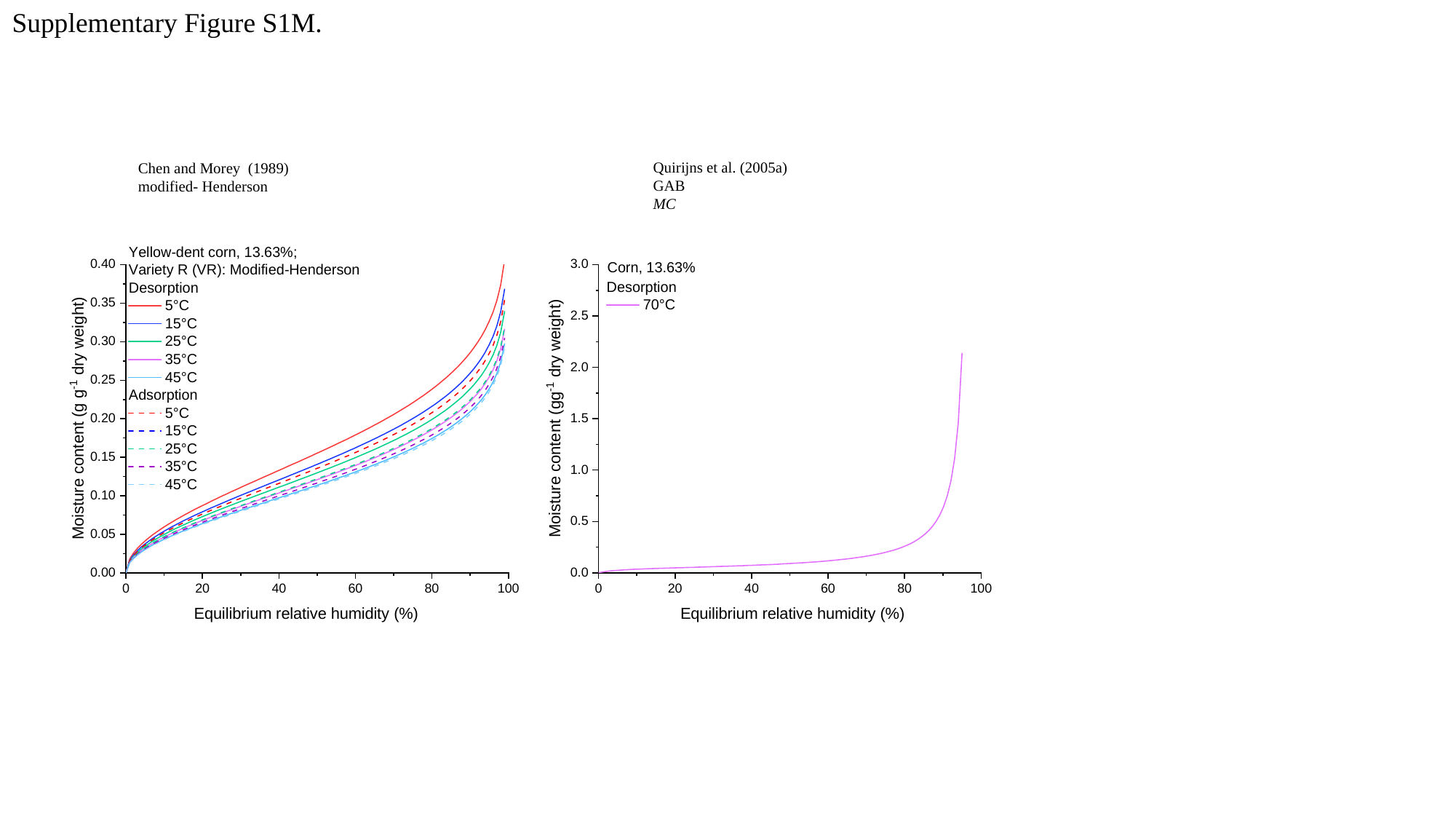

Supplementary Figure S1M.
Equilibrium relative humidity (%)
Equilibrium relative humidity (%)

## Slide 15
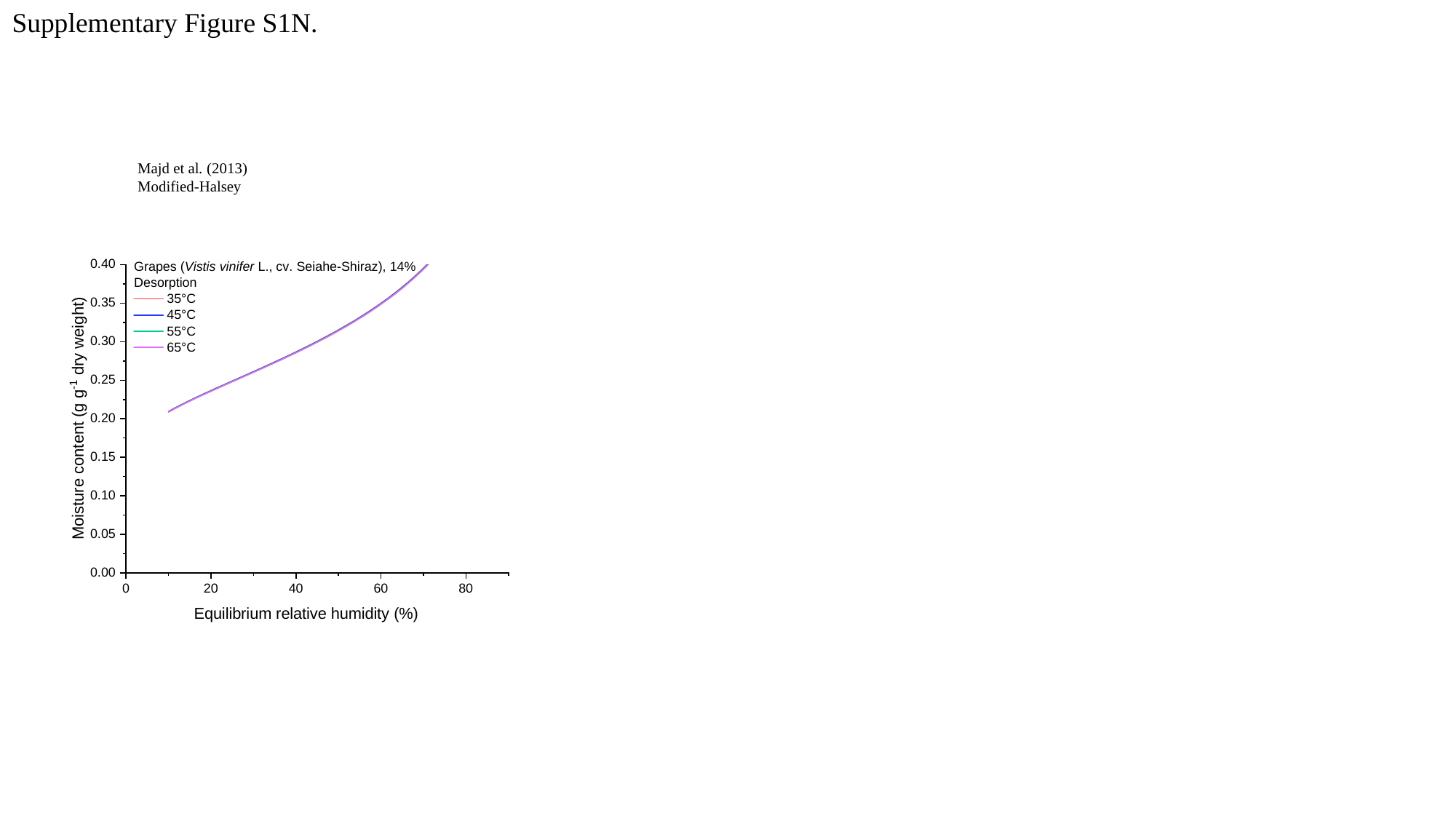

Supplementary Figure S1N.
Equilibrium relative humidity (%)

## Slide 16
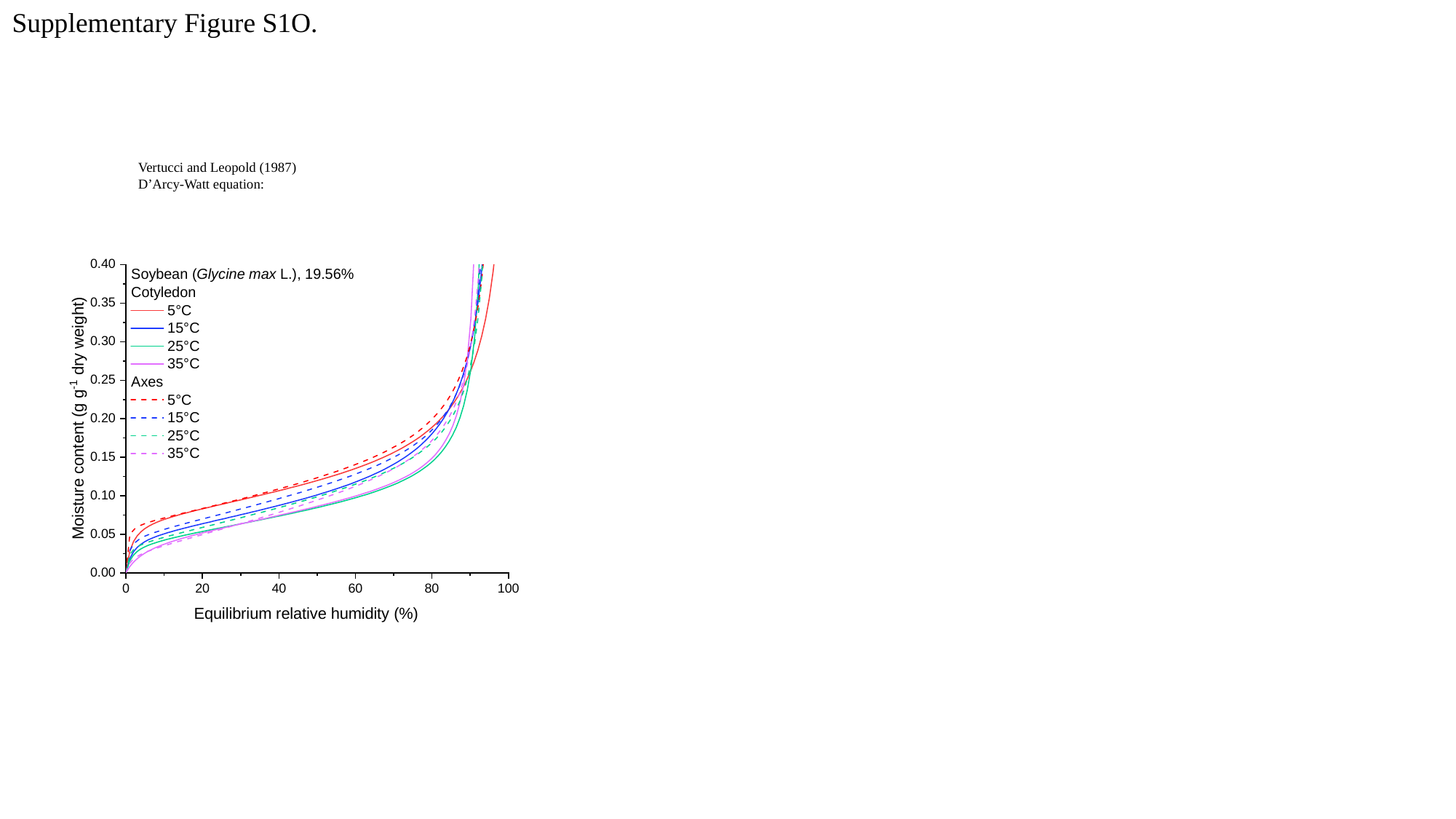

Supplementary Figure S1O.
Equilibrium relative humidity (%)

## Slide 17
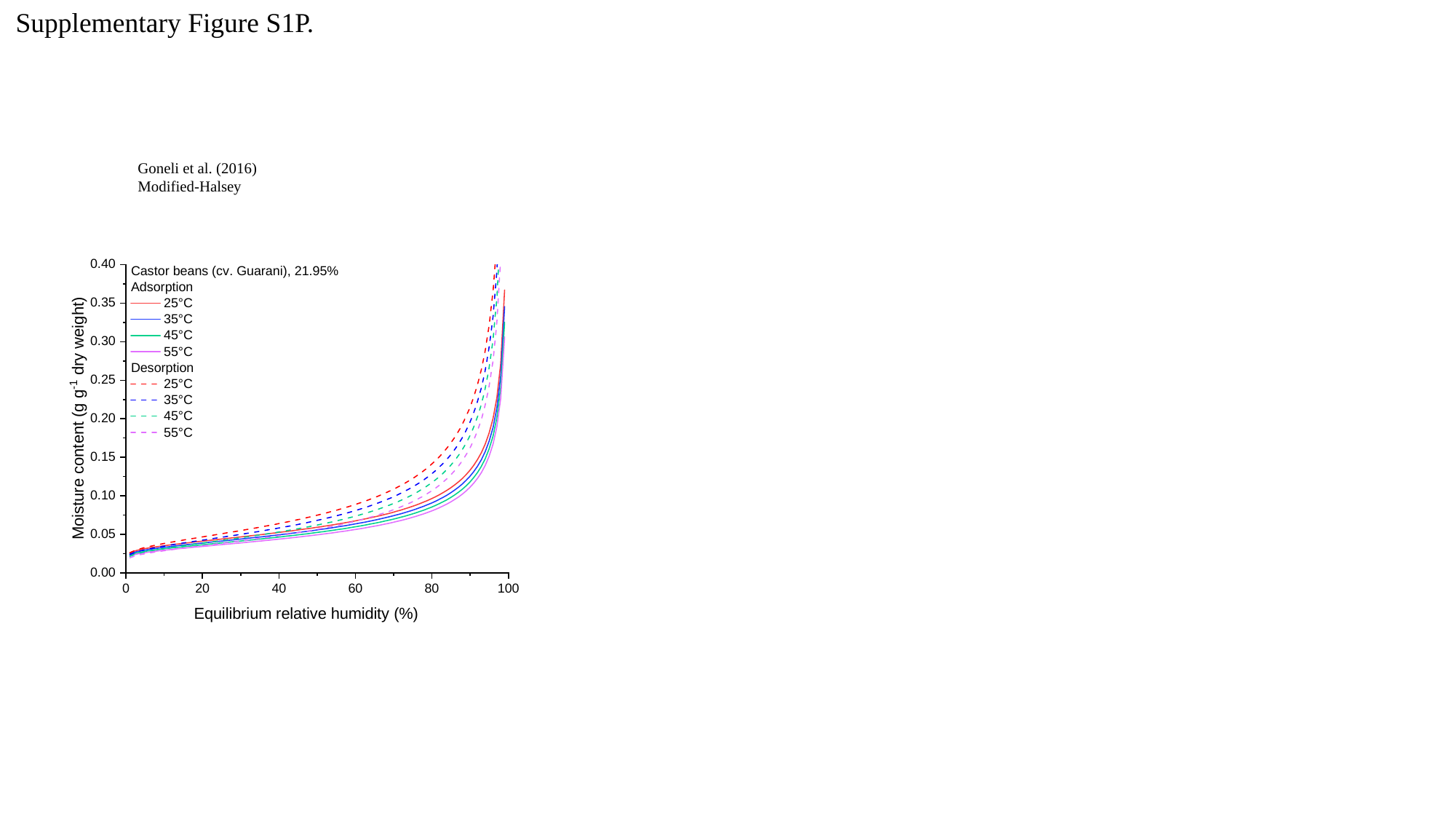

Supplementary Figure S1P.
Equilibrium relative humidity (%)

## Slide 18
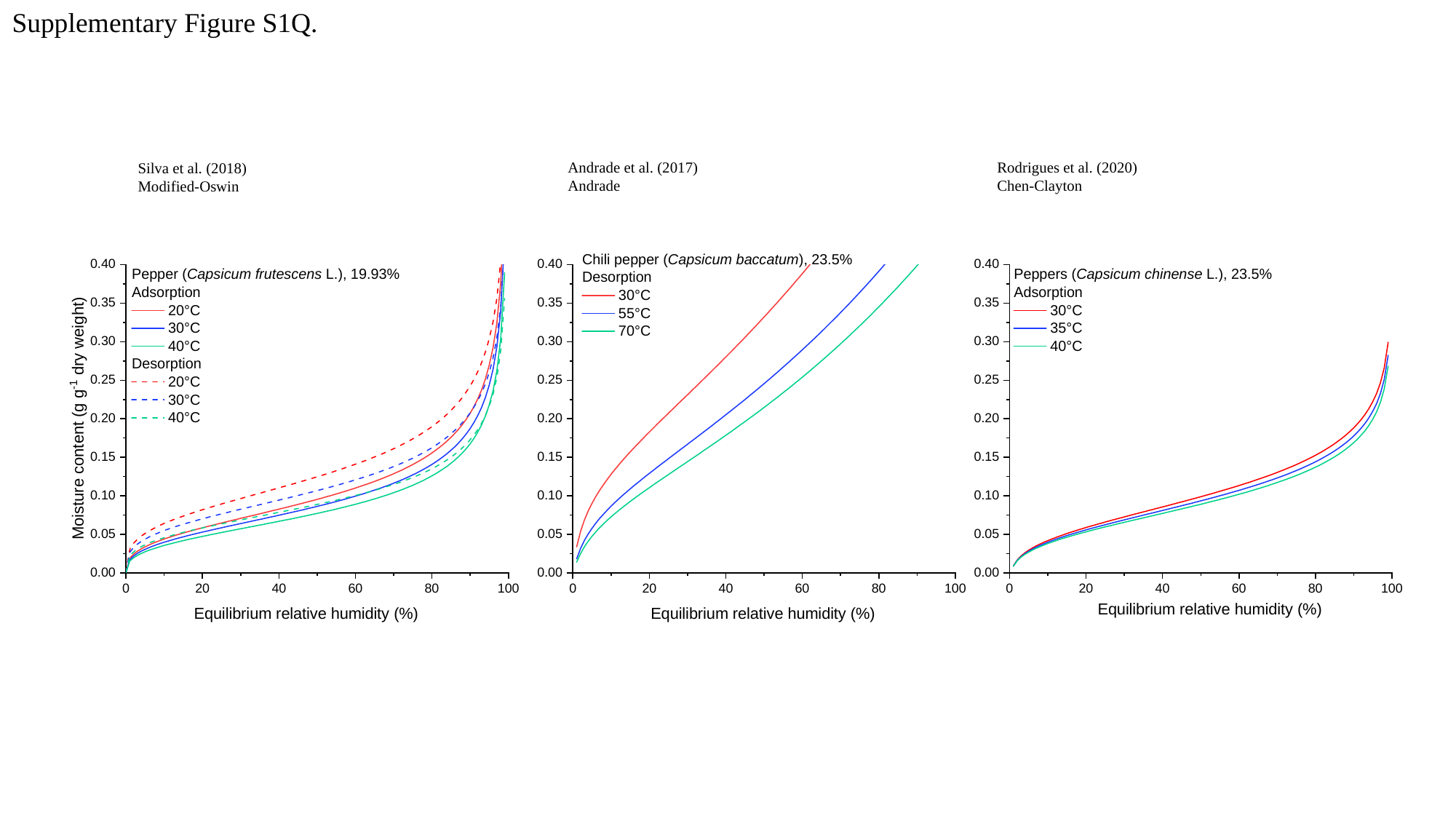

Supplementary Figure S1Q.
Equilibrium relative humidity (%)
Equilibrium relative humidity (%)
Equilibrium relative humidity (%)

## Slide 19
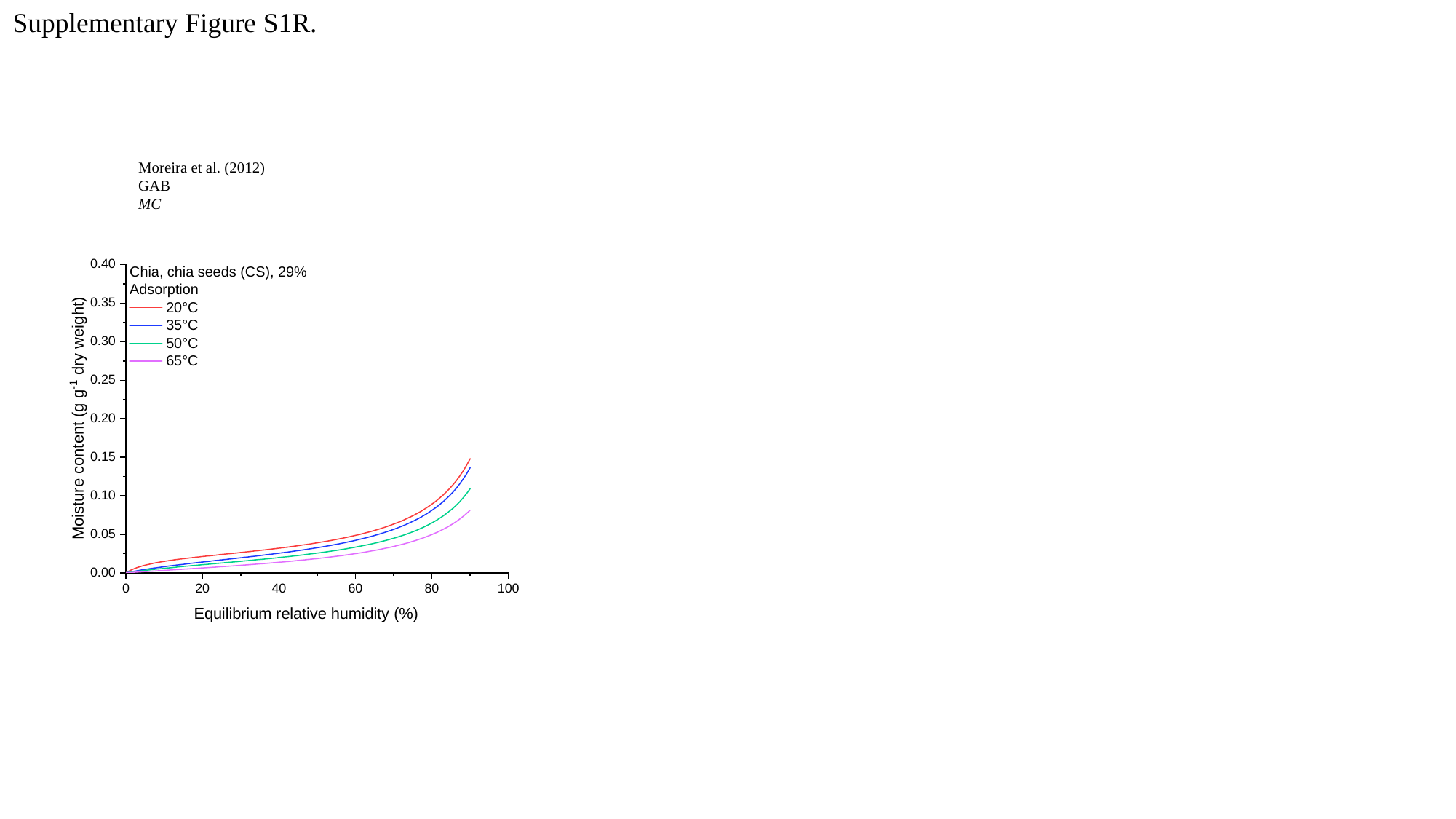

Supplementary Figure S1R.
Equilibrium relative humidity (%)

## Slide 20
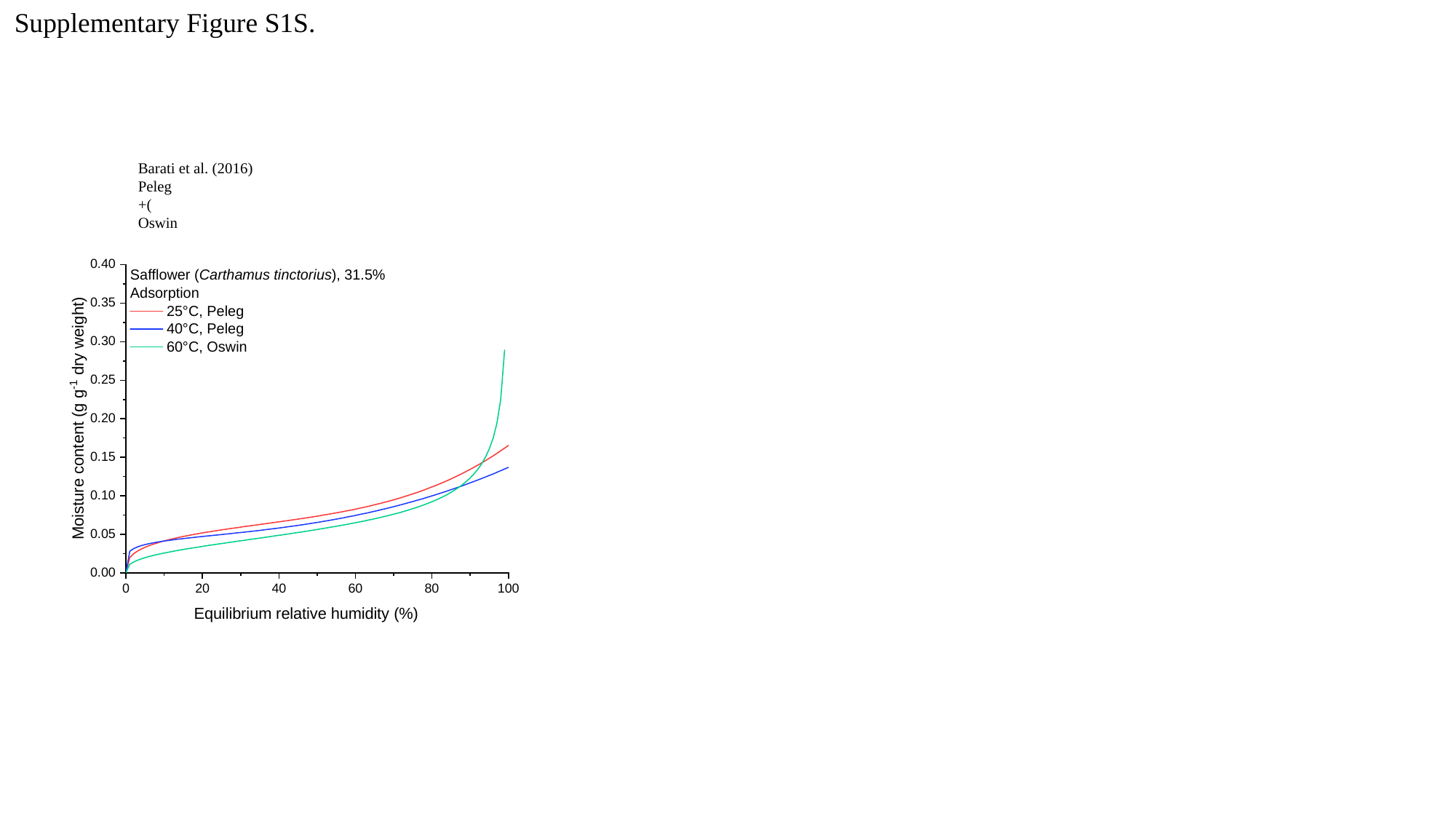

Supplementary Figure S1S.
Equilibrium relative humidity (%)

## Slide 21
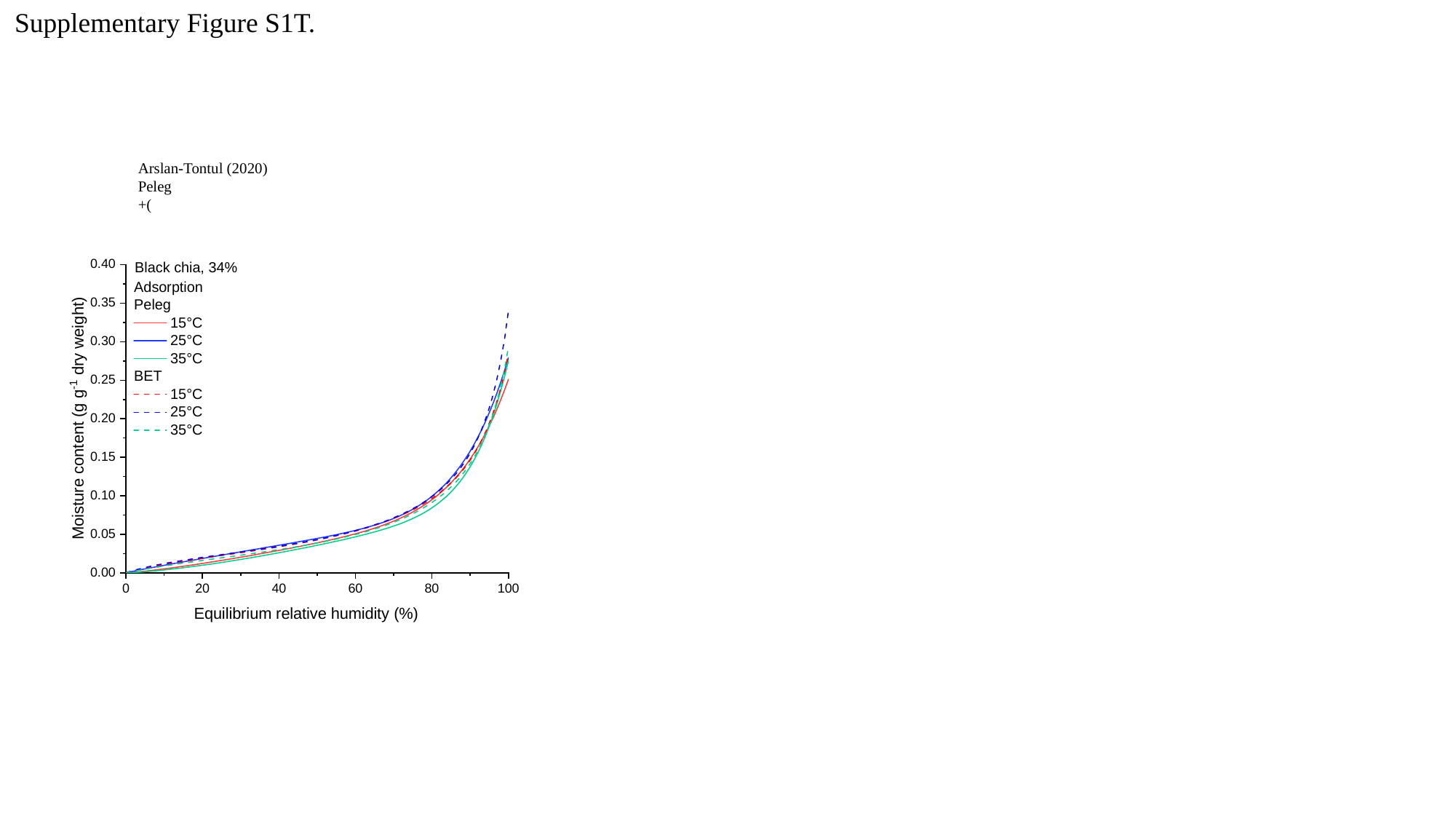

Supplementary Figure S1T.
Equilibrium relative humidity (%)

## Slide 22
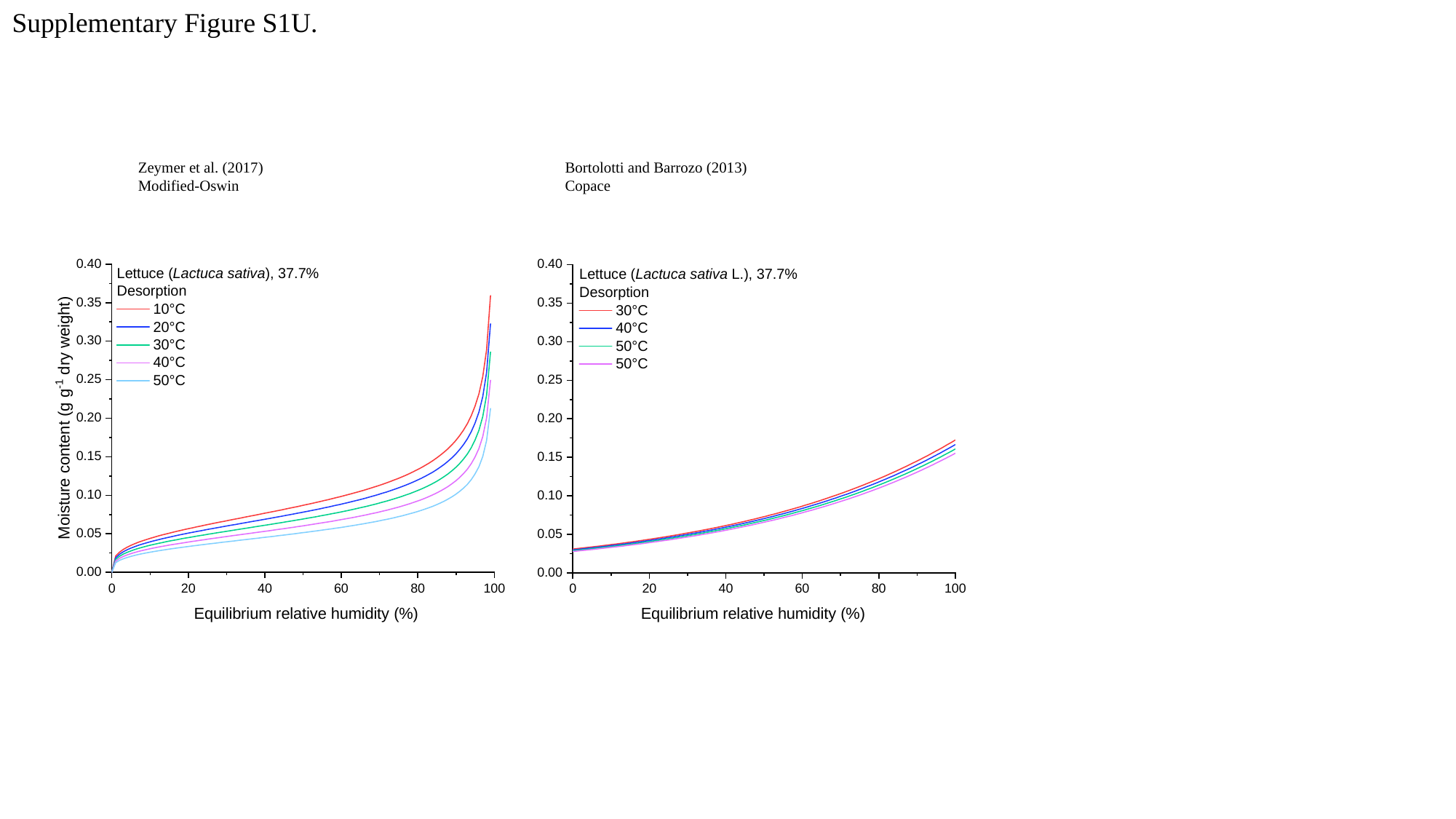

Supplementary Figure S1U.
Equilibrium relative humidity (%)
Equilibrium relative humidity (%)

## Slide 23
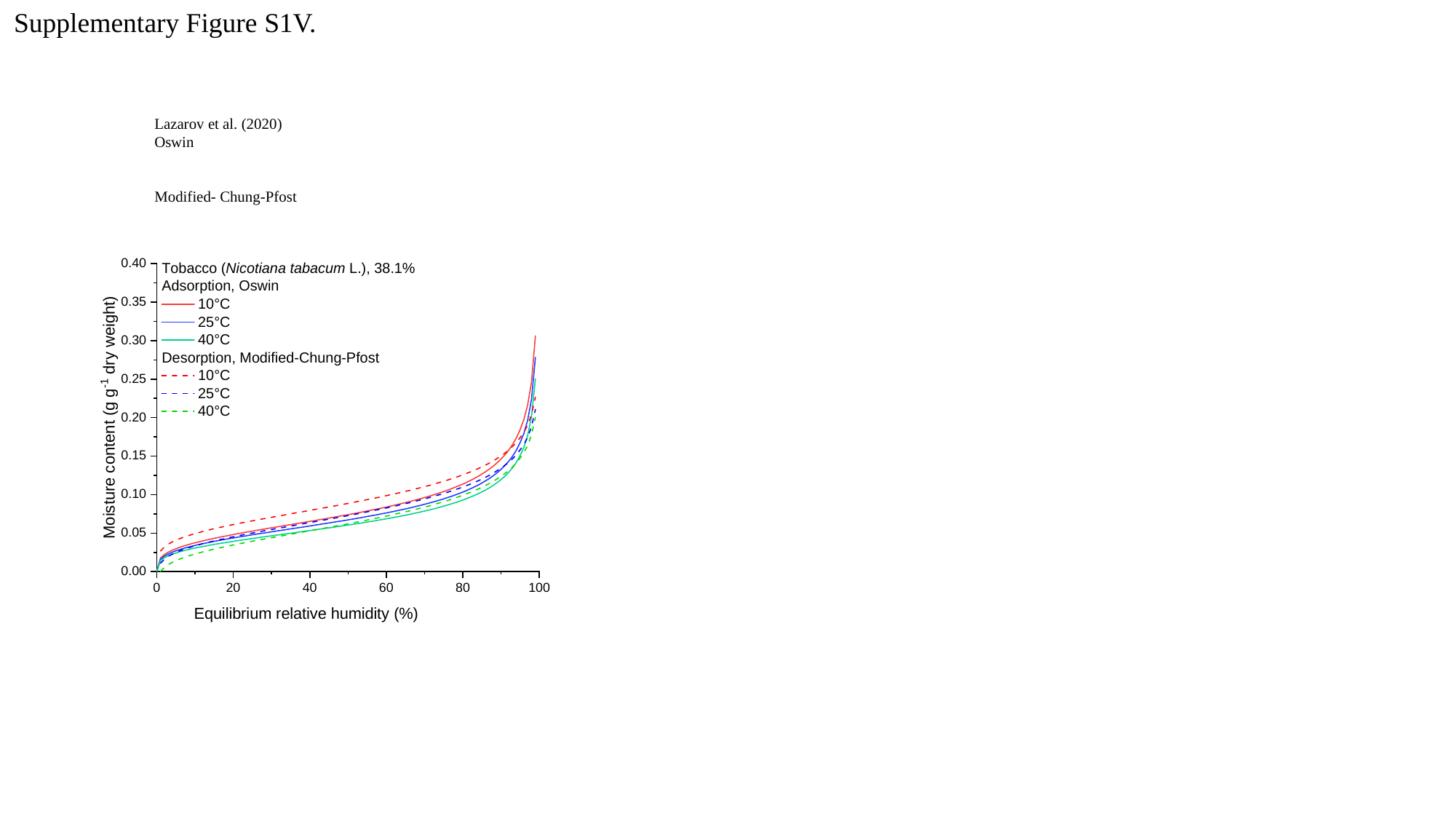

Supplementary Figure S1V.
Equilibrium relative humidity (%)

## Slide 24
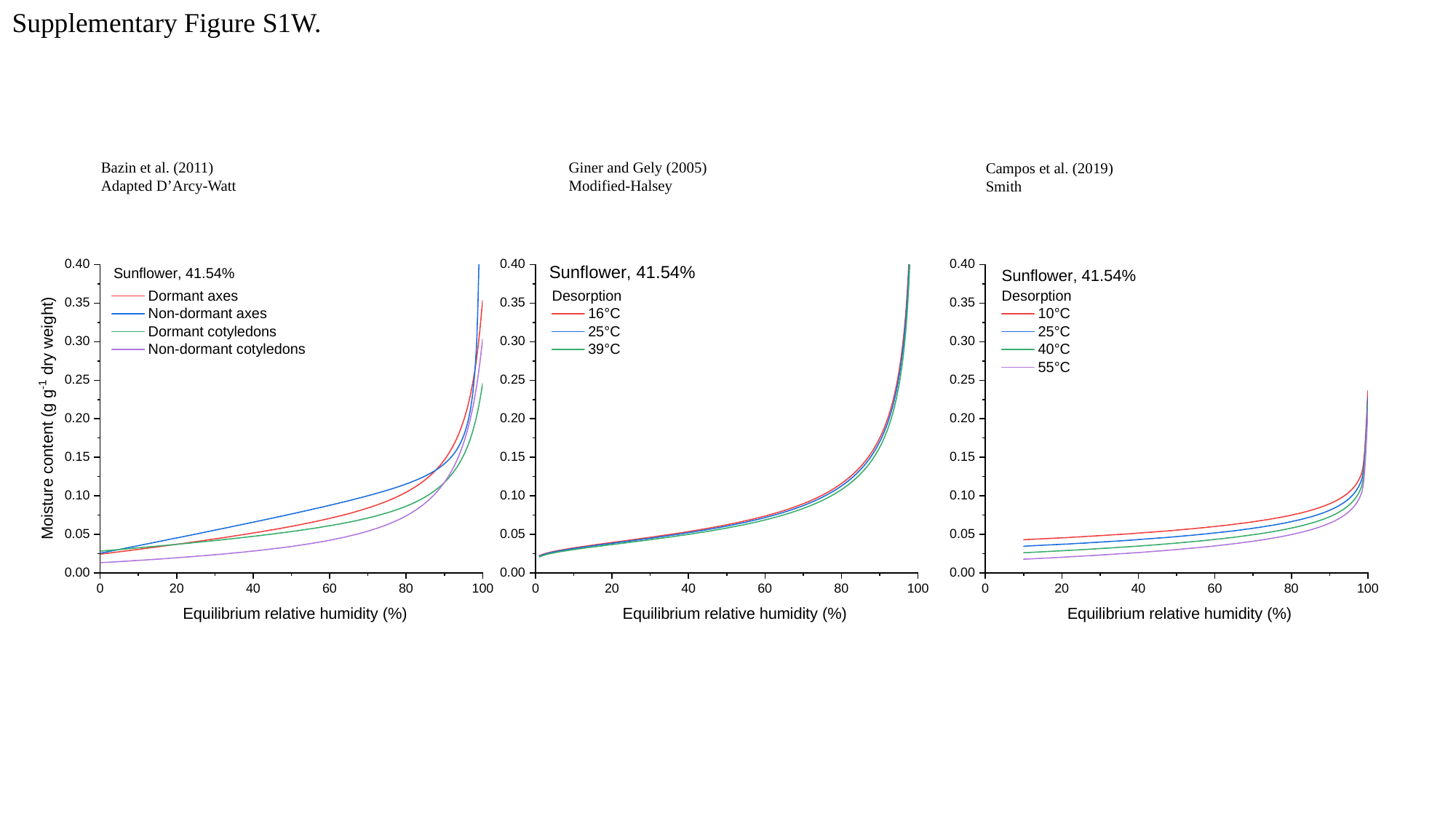

Supplementary Figure S1W.
Equilibrium relative humidity (%)
Equilibrium relative humidity (%)
Equilibrium relative humidity (%)

## Slide 25
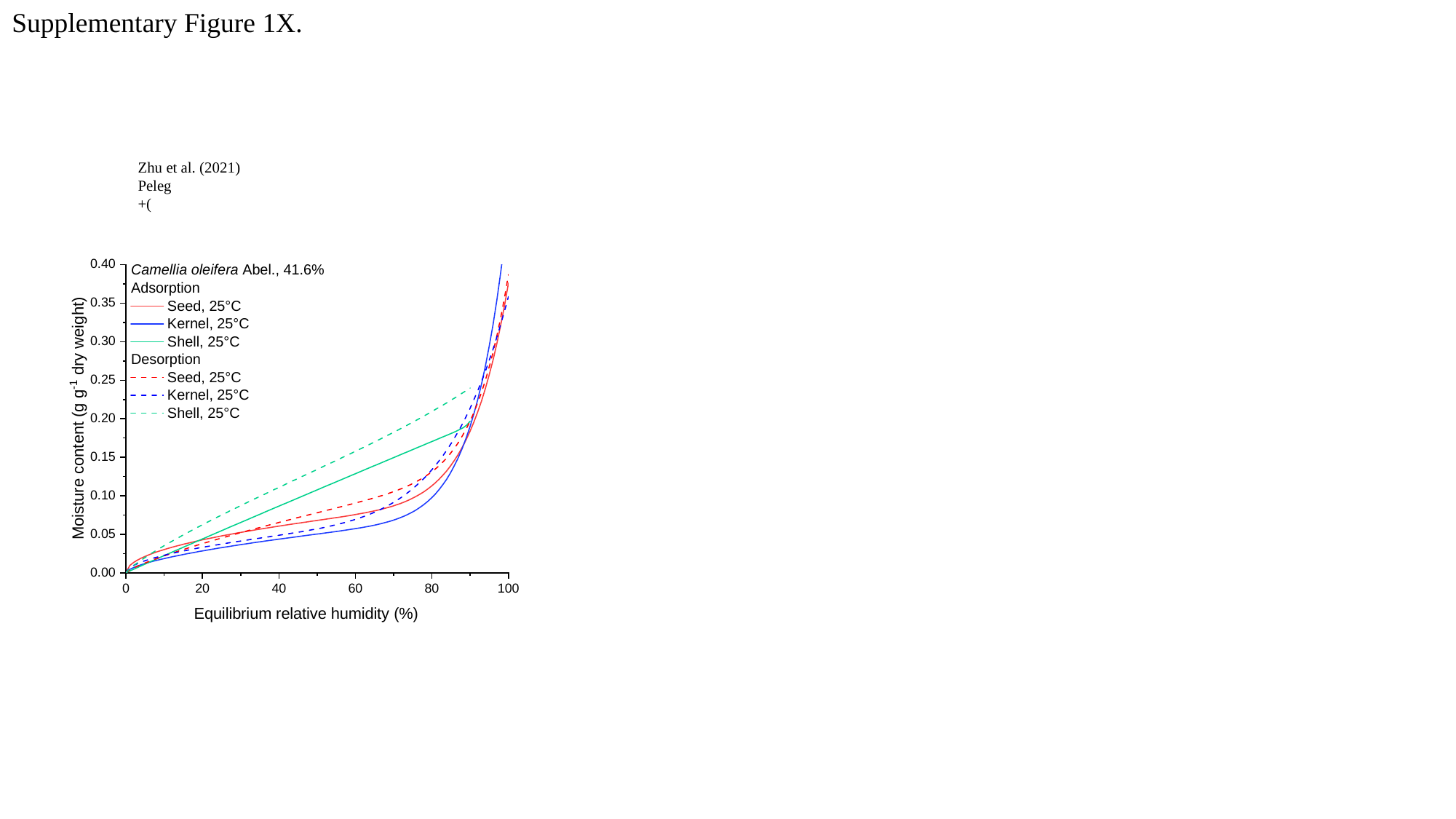

Supplementary Figure 1X.
Equilibrium relative humidity (%)

## Slide 26
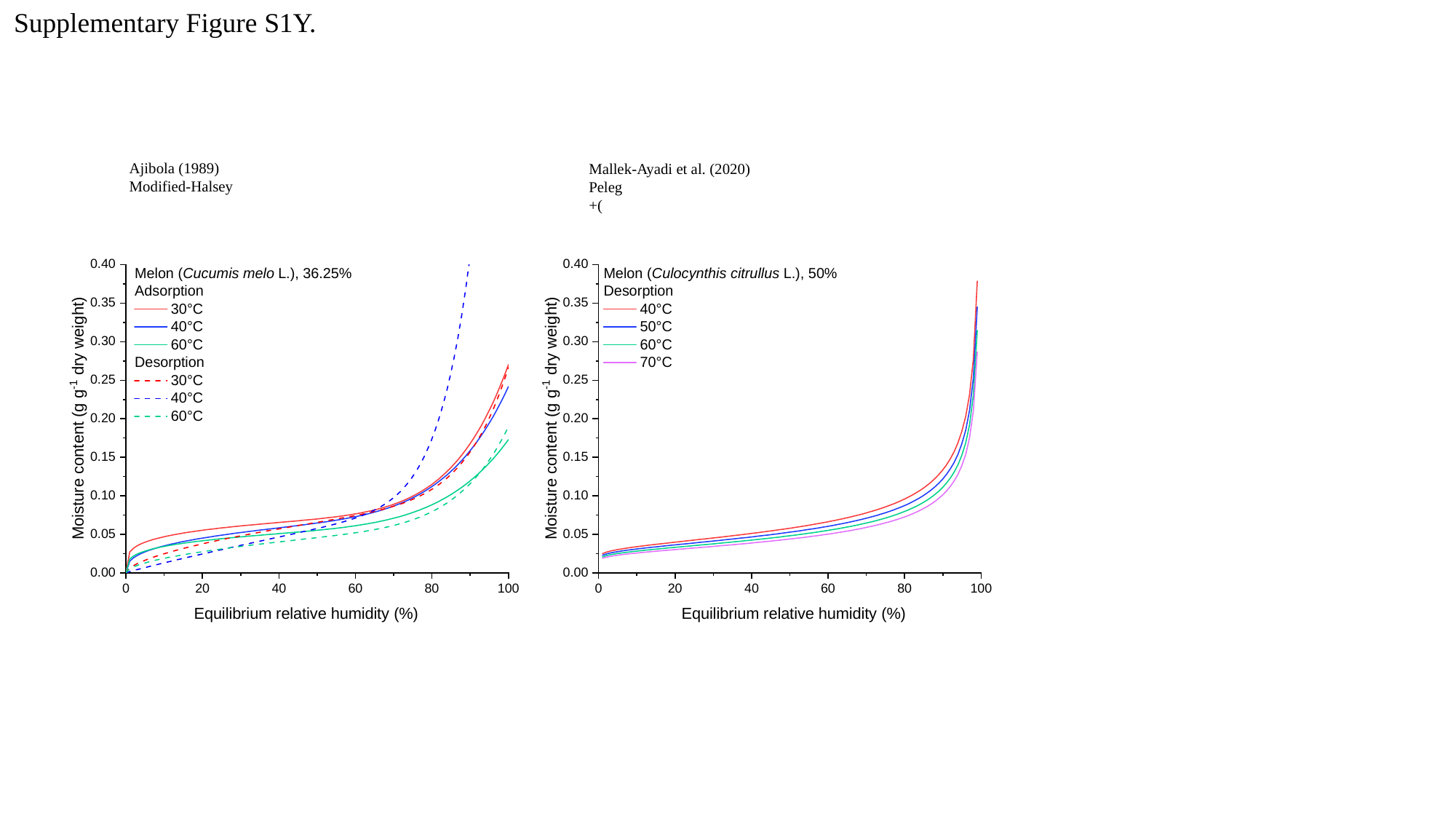

Supplementary Figure S1Y.
Equilibrium relative humidity (%)
Equilibrium relative humidity (%)

## Slide 27
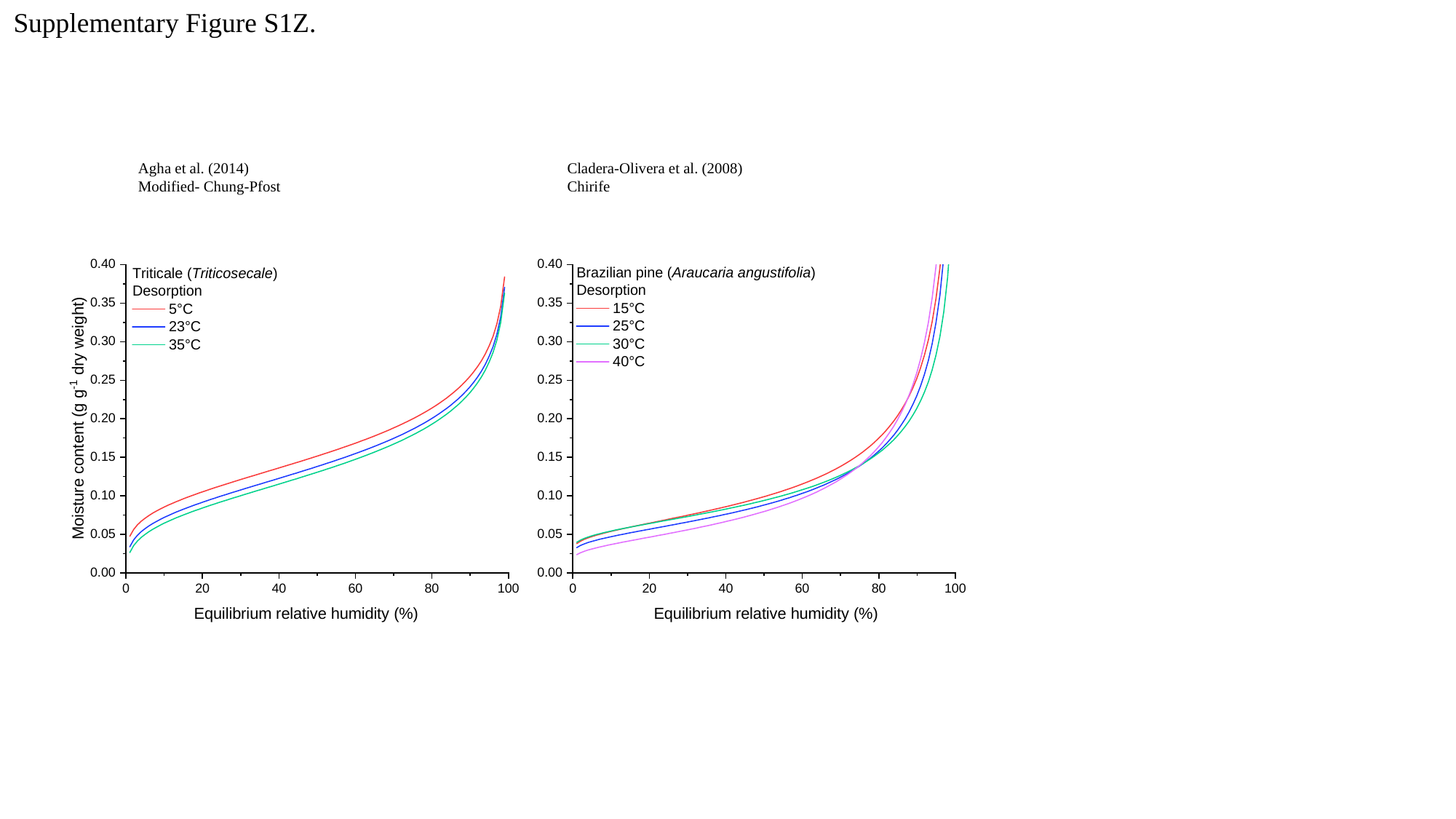

Supplementary Figure S1Z.
Equilibrium relative humidity (%)
Equilibrium relative humidity (%)
